# Efficacy of Quinine, Artemether-Lumefantrine and Dihydroartemisinin-Piperaquine as Rescue Treatment for Uncomplicated Malaria in Ugandan Children

**DOI:** 10.1371/journal.pone.0053772

**Published:** 2013-01-22

**Authors:** Adoke Yeka, James Tibenderana, Jane Achan, Umberto D'Alessandro, Ambrose O. Talisuna

**Affiliations:** 1 Uganda Malaria Surveillance Project, Kampala, Uganda; 2 Malaria Consortium, Kampala, Uganda; 3 Institute of Tropical Medicine, Antwerp, Belgium; 4 World Wide Antimalarial Resistance Network, University of Oxford/KEMRI/Wellcome Trust Research Programme, Nairobi, Kenya; London School of Hygiene and Tropical Medicine, United Kingdom

## Abstract

**Background:**

The treatment of falciparum malaria poses unique challenges in settings where malaria transmission intensity is high because recurrent infections are common. These could be new infections, recrudescences, or a combination of the two. Though several African countries continue to use quinine as the second line treatment for patients with recurrent infections, there is little information on its efficacy when used for rescue therapy. Moreover, such practice goes against the World Health Organisation (WHO) recommendation to use combination therapy for uncomplicated malaria.

**Methods:**

We conducted a nested, randomized, open label, three-arm clinical trial of rescue therapy in children 6–59 months old with recurrent malaria infection during 28 days post treatment with artemisinin combination treatment (ACT). Patients were randomly assigned to receive either quinine, artemether-lumefantrine (AL) or dihydroartemisinin-piperaquine (DHAPQ), and actively followed up for 28 days.

**Findings:**

Among 220 patients enrolled, 217 (98^.^6 %) were assigned an efficacy outcome and 218 (99^.^1 %) were assessed for safety. The risk of recurrent infection was significantly higher in patients treated with quinine (70 %, 74/110, HR = 3^.^9; 95 % CI: 2^.^4–6^.^7, p<0^.^0001) and AL (60%, 21/35, HR = 3^.^3; 95 % CI: 1^.^8–6^.^3, p<0^.^0002), compared to DHAPQ (25%, 18/72). Recrudescence tended to be lower in the DHAPQ (1%, 1/72) than in the quinine (7%, 8/110) or AL (6 %, 2/35) group, though it was not statistically significant. No serious adverse events were reported.

**Conclusion:**

Recurrent infections observed after the administration of an ACT can be successfully treated with an alternative ACT rather than with quinine.

**Trial Registration:**

Current Controlled Trials ISRCTN99046537

## Introduction

Most malaria endemic countries have deployed artemisinin combination therapy (ACTs) as first line regimens for the treatment of uncomplicated falciparum malaria [1]. ACTs are highly efficacious, well tolerated, reduce gametocyte carriage and could, if well used and deployed, delay the emergence and spread of antimalarial drug resistance [2], [3]. Where malaria transmission is intense, recurrent infections, i.e. peripheral infections (with or without fever) following the treatment of primary episodes, are a common occurrence [4], [5]. Though a recurrent infection may be either a recrudescence (same infection as the primary one) or a new infection, such information is not immediately available for managing the patient as the necessary molecular analysis of the parasite isolates is done later in a well-equipped laboratory. Patients with recurrent infections are usually given the second line treatment which should be safe and efficacious in case of resistance to the treatment used for the primary episode. In several sub-Saharan African countries, the second line treatment is oral quinine [1], [6], a policy not consistent with the current WHO recommendations to use combination therapy for uncomplicated malaria [1], [7]. There is little or no information available on ACTs used as rescue treatments. Therefore, a study aiming at establishing the safety and efficacy of two different ACTs when used as rescue treatment as compared to quinine, the treatment routinely used for this purpose, was carried out in Tororo, Uganda, an area of intense malaria transmission. Results are reported below.

## Methods

### Study design

This nested study was carried out in Tororo, Uganda, one of the sites participating in a large multi-centre trial (12 sites spread over 7 countries) on the safety and efficacy of four ACTs (artemether-lumefantrine-AL, amodiaquine-artesunate-ASAQ, dihydroartemisinin-piperaquine-DHAPQ, and chlorproguanil-dapsone and artesunate-CD+A). Details of the multi-centre trial are published elsewhere [8]. Briefly, children 6–59 months old at the time of inclusion were randomised and treated with one of the four study drugs, followed up actively for 28 days (first active follow up) and then passively for the next 6 months. After the first 28 days, if they experienced a second malaria episode, they were treated with the same ACT used for the first episode and actively followed up for 28 additional days (second active follow up). Each site tested three out of the four ACTs. In case of treatment failure during the first or second active follow up, patients were given the rescue therapy according to the prevailing national recommendations (quinine for most study sites). At Tororo, the site where this nested study was carried out, the study treatments for the primary episode were AL, CD+A and DHAPQ. Patients with late treatment failure (after day 3 of the follow up and up to day 28) during the first or second active follow up were recruited into the nested study and randomised to either quinine (the recommended second line) or an ACT- either AL or DHAPQ. Group allocation depended on previous treatment and different randomization processes were employed to achieve this. Children who got AL in the first study were randomized to either the QN or DHAPQ arm, those who got DHAPQ were randomised to the AL or QN arms and those who got CD+A were randomised to either the QN, AL and DHAPQ arm. The protocol for this trial and supporting CONSORT checklist are available as supporting information; see Checklist S1 and Protocol S1

### Study site

The nested study was conducted between December 2007 and April 2009 at Nagongera Health Centre, in Tororo district, Uganda, an area of intense perennial malaria transmission (annual entomological inoculation rate estimated at 562 infective bites per person per year) [9]. The main and nested studies were approved in Uganda by the Makerere University Faculty of Medicine Research and Ethics committee and by the Ugandan National Council for Science and Technology, and in Belgium by the Institutional Review Board of the Institute of Tropical Medicine and by the Ethical Committee of the University Hospital in Antwerp. The study approvals are available as supporting information; see File S2 and S3.

### Participants

Patients from the main trial were included in this nested study if they had late treatment failure (recurrent infection with or without fever) between day 4 and day 28 of the two active follow ups. The participant selection criteria in the main study have been reported elsewhere [1], [8]. Children experiencing an early treatment failure, severe malaria or having danger signs, or those with history of hypersensitivity to the study drugs were excluded and treated with parenteral quinine [1], [10], [11]. Caregivers were explained the study objectives and procedures, and asked to provide a written informed consent.

### Baseline Evaluation, Randomization and Treatment

**Figure 1:**
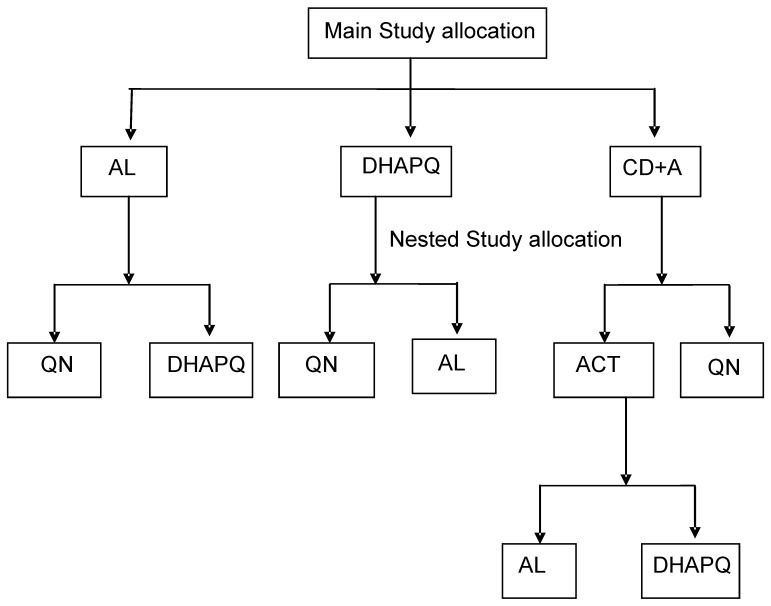
The study design.

At enrolment, patient's symptoms were evaluated. Temperature (axillary) and weight were measured and a focused physical examination was performed. A thick and thin blood smear for parasitaemia and a blood sample on filter paper (Whatman 3MM) for later molecular analysis were collected before treatment. Patients were randomly assigned to receive either quinine or one of the two ACTs, (AL or DHAPQ). For the latter, to avoid giving the same ACT administered for the primary episode as rescue treatment, patients having received AL as primary treatment were treated with DHAPQ as rescue treatment and vice versa; patients having received CD+A were randomized to one of the two ACTs (Figure 1). A randomization list was computer generated by a member of the project not directly involved in patient management; sequentially numbered, sealed envelopes containing the treatment assigned were prepared according to the randomization lists. The study nurse assigned treatment numbers sequentially and allocated treatment by opening the envelope corresponding to the treatment number. A second randomisation list was used to randomise patients initially treated with CD+A to either AL or DHAPQ. Only the study nurse had access to the sealed treatment randomization list though, given the variation in appearance, taste and dosing, the other research staff may have known to which arm children were assigned. Molecular analysis of the parasite isolates was carried out blindly, without knowing the treatment received by the patient. Supervised treatment allocation and administration of medications was performed by the study nurse who administered all medications orally as follows: AL (Coartem, Novartis, 20 mg artemether/120 mg lumefantrine tablets), administered according to the body weight as : one (5–14 kg), two (15–24 kg), or three (25–34 kg) tablets given twice daily for 3 days; DHAPQ (Eurartesim, Sigma-Tau, Italy, dihydroartemisinin (DHA) 20 mg+piperaquine phosphate (PQP) 160 mg and DHA 40 mg+PQP 320 mg tablets), given once daily for three days, at the standard dosage of 2^.^25 mg/kg and 18 mg/kg per dose of DHA and PQP, respectively, rounded up to the nearest ¼ tablet. Participants in the quinine arm received a seven day course of quinine sulphate as 10 mg/kg body weight per dose three times daily. Quinine sulphate was provided as 300 mg tablets (Rene Pharmaceutical, Kampala, Uganda); the quality of the drug was certified by the Uganda National Drug Authority. We used a pill cutter to ensure the tablet fractions were as close to the nearest ¼ tablet as possible. The administration of all doses was directly observed. Patients were kept for 30 minutes after treatment and the dose was re-administered if vomiting occurred. Patients who vomited persistently were given parenteral quinine. All patients were provided with a 3-day supply of paracetamol for the treatment of febrile symptoms. Patients with haemoglobin <10·0 g/dL were treated with ferrous sulphate for 14 days and given mebendazole if they were over one year of age and had not been treated in the previous 6 months. All patients who developed severe/complicated malaria during active follow-up were treated with parenteral quinine. Patients randomised to ACT who failed therapy were treated with quinine 10 mg/kg orally three times a day for 7 days. Patients who failed quinine therapy were treated with artesunate (2 mg/kg once a day) and clindamycin (10 mg/kg twice a day) for 7 days.

### Follow-up Procedures

At enrolment, children's parents or guardians were asked about use of other medications and presence of common symptoms. Axillary temperature and weight were recorded, and a physical examination performed. A blood sample for thick and thin blood smears, hemoglobin assessment and later molecular analysis (on filter paper) was collected by fingerprick. Similarly, at follow up visits scheduled for Days 1, 2, 3, 7, 14, 21 and 28, a standardized history was collected and physical examination performed. At each visit, including unscheduled ones, blood for thick blood smears and later genotyping was collected (by finger prick). Hemoglobin measurement was repeated on day 28 or the day of late clinical failure. Patients were encouraged to attend the clinic at any time if ill. Patients not attending the clinic at the scheduled visits were actively followed up at home.

### Laboratory Evaluations

Thick and thin blood smears were stained with 2% Giemsa for 30 minutes. Parasite density was determined by reading the thick blood smear and counting the number of asexual parasites per 200 white blood cells (WBCs), assuming a WBC count of 8,000/µl. Slides were considered negative if no parasite was detected after reading 100 high-powered fields. Presence of gametocytes was also recorded. Thin blood smears were reviewed for non-falciparum infections. Two microscopists independently read all slides and parasite densities were calculated by averaging the two counts. Readings with discordant results (difference in species diagnosis, difference in parasite density of >50%, or any difference that affected recruitment or study outcome) were re-examined by a third microscopist; the parasite density was calculated by averaging the two closest densities while the final parasite species was determined by the two concordant reads. Hemoglobin measurements were made using a portable spectrophotometer (HemoCue, Ängelholm, Sweden).

Molecular genotyping was carried out at the Institute of Tropical Medicine, Antwerp, Belgium, on samples collected from patients with late treatment failure to discriminate between a recrudescent and a new infection. Parasite deoxyribonucleic acid (DNA) was isolated from filter paper blood samples collected at enrolment and on the day of recurrent parasitemia using chelex extraction. Genotyping was done according to international recommendations [12]. The DNA was purified as previously described [13] and three polymorphic genetic markers were genotyped sequentially, starting with glutamate rich protein (GluRP), followed by merozoite surface protein-2 (MSP2), and ending with merozoite surface protein-1 (MSP1). Capillary electrophoresis was used for MSP2. Whenever a genetic marker showed a new infection, (no common allele between day of recurrent infection and day 0) this was taken as the final result and the analysis was stopped. For samples showing a recurrent infection, (at least one identical allele between day 0 and day of recurrent infection) the analysis was carried out until MSP1. If the latter showed also at least one identical allele between day of recurrent infection and day 0, then the infection was classified as a recrudescence [12]. All results were double read and discrepancies resolved. The InGenius Gel Documentation and Analysis system Standard Operating Procedure (SOP) is available as supporting information; see File S1.

### Objectives

The objectives of the study were to compare the safety and efficacy of quinine with that of two other ACT, i.e. AL or DHAPQ, when used as rescue treatment for late treatment failure (clinical or parasitological) following a primary episode of uncomplicated malaria.

### Outcomes – Efficacy

Treatment outcomes were classified according to the WHO guidelines for areas of intense transmission as adequate clinical and parasitological response (ACPR), early treatment failure (ETF), late clinical failure (LCF), and late parasitological failure (LPF) [11]. Failure was defined as the sum of ETF, LCF and LPF. Patients were not assigned an efficacy outcome for the following reasons: 1) administration of antimalarial drugs outside the study protocol; 2) withdrawal of consent; and 3) loss to follow-up.

Day 28 treatment outcomes were adjusted to distinguish recrudescent and new infections using molecular genotyping based on GLURP, MSP2 and MSP1 polymorphisms. Recrudescence was defined by a paired sample having at least one identical allele present on each of the three loci. A new infection was classified as infection with all alleles different in at least one locus. When no amplification was observed for either of the paired samples, the outcome was classified as indeterminate.

The primary efficacy endpoint was the clinical and parasitological outcome at day 28, unadjusted and adjusted by genotyping. Secondary efficacy endpoints included, fever and parasite clearance, gametocytaemia (prevalence and density) at day 7, 14, 21 and 28, haemoglobin changes between Day 0 and Day 28 or the day of treatment failure, and incidence of adverse events.

### Outcomes - safety

Safety outcomes included risk of adverse events. At each follow-up visit, any new or worsening event and laboratory results were assessed. An adverse event was defined as any untoward medical occurrence, irrespective of its relationship to the study medications (Guidance for Industry Good Clinical Practice: Consolidated Guidance [ICH E6], April 1996). All events were graded by severity (none, mild, moderate, severe, life-threatening) and relationship to study treatment was classified as none, unlikely, possible, probable, or definite using WHO (toxicity grading scale for determining the severity of adverse events) and National Institute of Health (paediatric toxicity tables, May 2001) guidelines. A serious adverse event was defined as any event that resulted in inpatient hospitalization, death, life threatening experience, persistent/significant disability, or specific medical/surgical intervention to prevent serious outcome.

### Sample size

The study was designed to test the hypothesis that treatment with AL or DHAPQ would change the risk of recurrent parasitaemia (unadjusted by genotyping) after 28 days of treatment compared to Quinine. The main study included 510 children (170 per arm) with uncomplicated malaria. It was expected that about half of them (230) between days 4 and 28 would need rescue treatment as this is an area of high transmission [14] and be randomized to either quinine or one of the two ACT. Assuming 50% of children in the ACT arms would experience an unadjusted treatment failure compared to 60% or more in the quinine arm, we would be able to detect, with 80% power and 5% level of significance, a significant difference between the treatment groups.

### Statistical Methods

Efficacy and safety data were evaluated using an intention-to-treat analysis, including all patients with falciparum malaria randomized to one of the study treatments. Data were double-entered (EpiInfo 6·04®, Centers for Disease Control and Prevention, Atlanta, GA, USA), verified, and analyzed using Stata version 10·0 (Stata Corporation, College Station, TX, USA). Parasite densities were normalized using logarithmic transformation. Risks of treatment failure were estimated using the Kaplan-Meier product limit formula. Data were censored for patients who did not complete follow-up or were reinfected with non-falciparum species. For the polymerase chain reaction (PCR)-adjusted ACPR, new *Plasmodium falciparum* infections detected on the basis of genotyping were also censored. Comparisons of treatment efficacy were made using a Cox proportional hazards model. We applied a Bonferroni correction to mitigate the probability of observing a significant difference by chance. With three comparisons, a p-value less than 0.017 (0.05/3) was considered significant.

## Results

### Patient Characteristics

**Figure 2:**
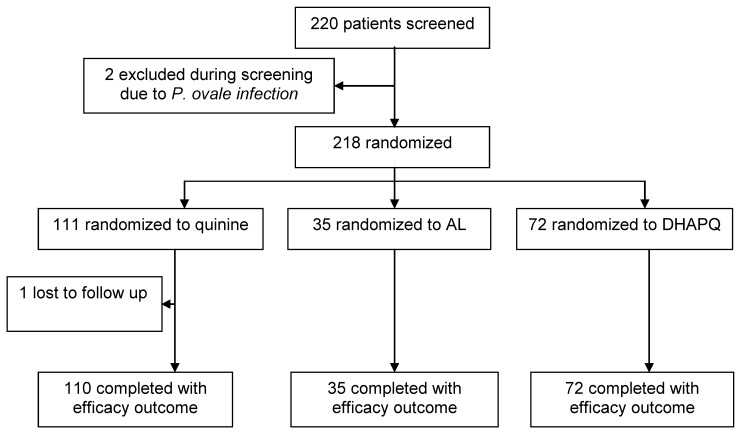
Trial profile.

Among the 220 eligible patients, two were excluded because they had a *P. ovale* infection and one was lost to follow up (Figure 2). The remaining 217 patients had an efficacy outcome. The three treatment group were comparable for the baseline characteristics though children randomised to AL seemed to have a lower parasite density and less fever (Table 1).

**Table 1:** Baseline characteristics of patients with efficacy outcomes.

Characteristic	Treatment group*		
	Quinine (n = 111)	AL (n = 35)	DHAPQ (n = 72)
Female (%)†	50 (46%)	18 (51%)	33 (46%)
Age in months, median (IQR)	23 (33 -17)	21 (30 -15)	24 (32–18)
Fever (%)	39.00	17.60	39.40
Parasite density per µL, geometric mean (95% CI)	8107 (5727–11475)	2800 (1370–6314)	6601(4216–10335)
Hemoglobin gm/dL, mean (SD)	10^.^73 (1^.^27)	10^.^57 (1^.^41)	10^.^835 (1^.^34)
Gametocytes at day 0 (%)	0 (0.0)	0 (0.0)	0 (0.0)

* AL = Artemether- lumefantrine. DHAPQ = Dihydroartemisinin piperaquine.

† Fever defined as temperature ≥37.5°C.

### Primary efficacy outcomes

**Figure 3:**
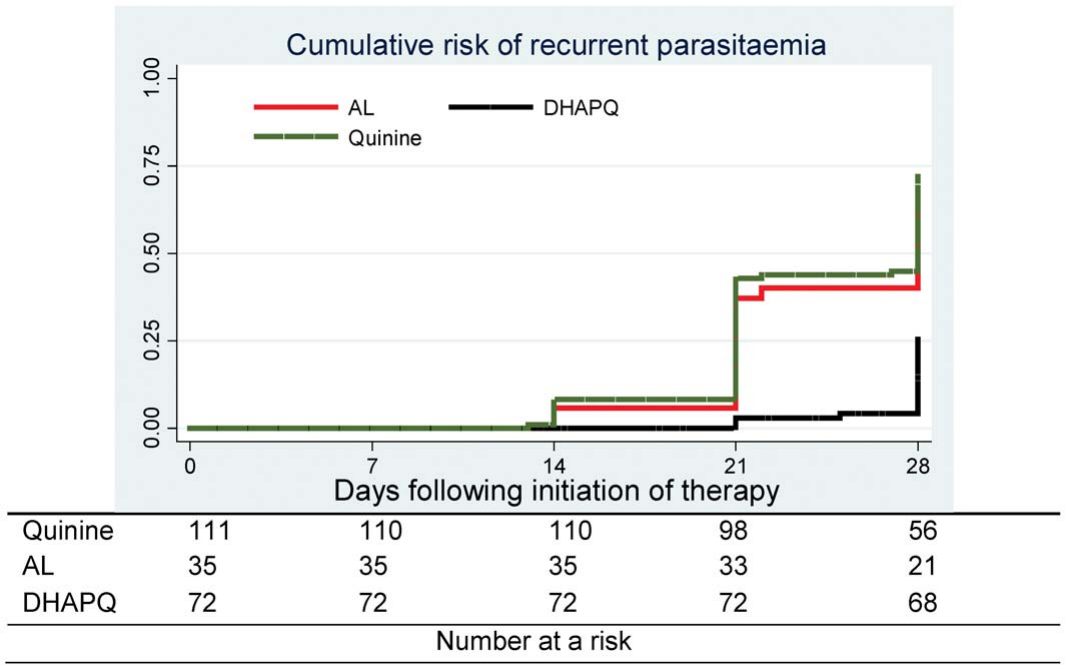
Cumulative risk of recurrent parasitaemia.

All 217 patients with efficacy outcomes were included in the analysis. No ETF was detected (Table 2). Almost 70% (74/110) of children in the quinine arm had a recurrent infection as compared to 60% (21/35) in the AL and 25% (18/72) in the DHAPQ arms (Table 2). Both quinine (HR = 3^.^9; 95%CI: 2^.^4–6^.^7)(p<0^.^0001) and AL (HR = 3^.^3; 95%CI: 1^.^8–6^.^3)(p<0^.^0002) had a significantly higher risk of recurrent infection as compared to DHAPQ (Table 3). No significant difference in recurrent infection was found between quinine and AL (p = 0.4) (Table 3). Most recurrent infections were identified as new infections. Though the PCR-adjusted treatment failure tended to be lower in the DHAPQ (1%, 1/72) than in the quinine (7%, 8/110) and AL (6 %, 2/35) groups, it did not reach statistical significance (Table 3). The rate of recurrent infections was lower and occurred later in the DHAPQ treatment group (Figure 3). The baseline parasite density and treatment allocation for the primary malaria episode had no influence on treatment outcome (data not shown).

**Table 2:** WHO treatment outcomes after 28 days of follow-up.

Treatment outcomes	Treatment group*		
	Quinine (n = 111)	AL (n = 35)	DHAPQ (n = 72)
Lost to follow-up (no treatment outcome)	1	0	0
Early treatment failure (ETF)	0	0	0
Late clinical failure (LCF)	31 (28%)	7 (20%)	6 (8%)
Late parasitological failure (LPF)	43 (39%)	14 (40%)	12 (17%)
Adequate clinical and parasitological response (ACPR)	36 (33%)	14 (40%)	54 (75%)
All failures			
Overall	74 (67%)	21 (60%)	18 (25%)
New infection.	62 (56%)	16 (46%)	15 (21%)
Recrudescence	8 (7%)	2 (6%)	1 (1%)
Genotyping unsuccessful	4 (4%)	3 (9%)	2 (3%)

* AL = Artemether- lumefantrine. DHAPQ = Dihydroartemisinin piperaquine.

**Table 3:** Estimates of comparative efficacy.

Risk category	28 day risk of treatment failure, % (95% Confidence Interval)	Hazard Ratio (95% CI)	p-value
* **Unadjusted by genotyping**			
Quinine vs AL	66^.^7 (59^.^0–75^.^0) vs 60^.^0 (43^.^0–76^.^0)	1^.^23 (0^.^78–2^.^00)	0.398
Quinine vs DHAPQ	66^.^7 (59^.^0–75^.^0) vs 25^.^0 (15^.^0–35^.^0)	3^.^98 (2^.^37–6^.^68)	<0.0001
AL vs DHAPQ	60^.^0 (43^.^0–76^.^0) vs 25^.^0 (15^.^0–35^.^0)	3^.^32 (1^.^76–6^.^26)	0.0002
† **Adjusted by genotyping.**			
Quinine vs AL	7^.^0 (2^.^0–12^.^0) vs 6^.^0 (0^.^1–12^.^0)	1^.^30 (0^.^27–6^.^22)	0^.^739
Quinine vs DHAPQ	7^.^0 (2^.^0–12^.^0) vs 1^.^0 (0^.^1–4^.^0)	2^.^09 (0^.^25–17^.^43)	0^.^497
AL vs DHAPQ	6^.^0 (0^.^1–12^.^0) vs 1^.^0 (0^.^1–4^.^0)	1^.^62 (0^.^14–18^.^31)	0^.^697

* episodes with no outcomes and recurrent parasitemia/malaria caused by non-falciparum species censored.

† episodes with no outcomes, recurrent parasitemia/malaria caused by non-falciparum species, and new *P. falciparum* infections Censored.

### Secondary efficacy outcomes

Patients treated with DHAPQ experienced less rapid resolution of fever compared to those treated with AL. However, by Day 3 fever had resolved in the majority of patients, regardless of treatment group. The percentage of children with parasitaemia at Day 2 was significantly lower in the ACT groups as compared to quinine, and among the ACT, DHAPQ performed significantly better than AL (Table 4). Nevertheless, at Day 3, none of the children treated with an ACT had a detectable parasitaemia.

**Table 4:** Secondary outcomes.

	Treatment group		
Category.	Quinine (n = 111)	AL (n = 35)	DHAPQ (n = 72)
**Fever clearance** *			
- Fever on day 1§	65 (58^.^6%)	14 (40^.^0%)	48 (66^.^7%)
- Fever on day 2	33 (29^.^7%)	21 (20^.^0%)	15 (20^.^8%)
- Fever on day 3	5 (4^.^5%	2 (5^.^7%)	4 (5^.^6%)
**Parasite clearance**			
- Parasitemia on day 2± ∥ §	56 (50^.^5%)	5 (14^.^3%)	2 (2^.^8%)
- Parasitemia on day 3	2 (1^.^8%)	0 (0^.^0%)	0 (0^.^0%)
a **Appearance of gametocytes not present on day 0**			
- Days 1–28	0 (0^.^0%)	0 (0^.^0%)	0 (0^.^0%)
**Hemoglobin (Hb) recovery** †			
- Mean increase (SD) in Hb (g/dL)	1^.^08 (1^.^4)	0^.^93 (1^.^7)	0^.^80 (1^.^7)
**Adverse events days 1–28**			
Cough	67 (60^.^4%)	20 (57^.^1%)	47 (65^.^3%)
Abdominal pain∥ §	0 (0^.^0%)	0 (0^.^0%)	5 (2.0%)
Anorexia	13 (11^.^7%)	3 (8^.^6%)	12 (16^.^7%)
Vomiting	1 (0^.^9%)	2 (5^.^7%)	3 (4^.^2%)
Weakness/malaise	4 (3^.^6%)	0 (0^.^0%)	1 (1^.^4%)
Diarrhoea	11 (9^.^9%)	2 (5^.^7%)	1 (1^.^4%)
**Serious adverse event**	0 (0^.^0%)	0 (0^.^0%)	0 (0^.^0%)

* Subjective fever over previous 24 hours or temperature ≥37^.^5°C.

† Change in Hb from day 0 to day 28 or day of clinical failure.

a Patients with gametocytes present on day 0 not included.

± Quinine vs AL, p<0^.^05.

∥ Quinine vs DHAPQ, p<0^.^05.

§ AL vs DHAPQ, p<0^.^05.

Adverse events were of mild or moderate severity and most of them were consistent with malaria symptoms. Cough, anorexia and diarrhoea were the most common, followed by abdominal pain, weakness and vomiting (Table 4). Abdominal pain was reported only among children treated with DHAPQ while weakness occurred more frequently in the quinine group. Children treated with ACTs had also a higher risk of vomiting as compared to quinine (Table 4). No serious adverse event was observed.

The proportion of children with anaemia (haemoglobin of <10·0 g/dL as per the Integrated Management of Childhood Illness guidelines) decreased in all treatment groups between Day 0 and Day 28 (quinine 24^.^3% to 9^.^8%, AL 25^.^7% to 5^.^6%, DHAPQ 18^.^3% to 6^.^1%). The mean haemoglobin increase between Day 0 and Day 28 was similar in the three groups (Table 4). No gametocytes were observed at enrolment or during follow-up.

## Discussion

The risk of recurrent infection at Day 28 after rescue treatment was significantly lower in the DHAPQ arm as compared to the quinine and AL arms. However, most infections were new so the efficacy of the three rescue treatments was not different when adjusted by genotyping, though there was a tendency towards a higher efficacy of DHAPQ.

In this study, quinine was administered under direct supervision and showed excellent efficacy. However, in a recent study carried out in Uganda, effectiveness of oral quinine was significantly lower than that of AL [15], a finding probably due to the less than optimal adherence of the patients to its difficult dosing schedule. Poor adherence to quinine is a well known problem, mainly due to the long duration of treatment and the common occurrence of cinchonism, a phenomenon characterised primarily by tinnitus, nausea, and dizziness [16]–[19]. Nevertheless, when administered under direct supervision as in the current study, quinine's efficacy is excellent. High quinine efficacy has similarly been observed in *in vitro* studies in Africa [20], [21], though resistance has been reported [22], [23].

Recent guidelines advocate for the use of quinine in combination with clindamycin or a tetracycline [1]. Quinine combined with tetracycline has been used in Southeast Asia as a second line treatment for uncomplicated falciparum malaria. When correctly used, the combination reliably clears parasites and fever. Nevertheless, treatment adherence is affected by the cinchonism adverse effects [24], [25]. Clindamycin combined with quinine is safe and effective in adults and children with multidrug-resistant *P. falciparum* malaria [25], [26]. This combination may be of particular value in children and pregnant women, in whom tetracyclines are contraindicated [26]. However, the combination is often not available or affordable in most endemic countries.

The ACTs (AL and DHAPQ) were highly efficacious for the treatment of recurrent falciparum malaria. AL has shown good efficacy in repeated treatments of recurrent *P. falciparum* malaria in other trials [27], [28]. Similarly, DHAPQ has shown good efficacy as rescue treatment for recurrent *P. falciparum* infections following 7-day treatments with combinations of quinine, artesunate or clindamycin in pregnancy [29]. Patients' adherence to ACTs is an equally important factor to ensure treatment efficacy, and to reduce the risk of selecting resistant parasites. Under conditions of routine clinical practice, adherence to AL was found to be suboptimal (64.1%) in Kenya [30], and low (38.7%) in Ethiopia [31]. In Uganda, full adherence to subsidised over the counter treatment with AL was only 13.2% [32]. Treatment with ACTs leads to the rapid clearance of certain symptoms associated with malaria illness, with the possibility that the patient would discontinue the treatment prematurely.

The cumulative risk of recurrent parasitaemia was higher after the second week of follow up. Similar findings have been observed in areas of intense malaria transmission in Uganda [5], [10], [14], [33]–[35] and could be explained by residual drug levels [36], [37]. The risk of recurrent parasitaemia was similar between patients treated with quinine and AL, and much lower in the DHAPQ group, a difference mainly due to new infections rather than recrudescences and explained by differences in pharmacokinetics between quinine and the non-artemisinin partner drugs. Piperaquine has a much longer elimination half-life [38] than lumefantrine [39], [40], which also may explain the lower mean parasite density in patients retreated with AL as they could have had persisting low-level piperaquine activity.

Historically, effective treatment – a vital component of an effective malaria control strategy- was hampered by the serial development of resistance to the most commonly used drugs. In the early 2000 s, the World Health Organization (WHO) recommended the adoption of ACTs for the treatment of non-complicated malaria. Most African countries now recommend ACTs as the first line regimen for treating uncomplicated malaria. Unfortunately, this effective treatment option is now under threat as recent reports from South East Asia suggest emerging resistance to the artemisinin derivatives [41]–[45]. The enormous public health impact, if artemisinin resistance were to arrive in Africa, is compounded by significant reductions in the systematic evaluation of antimalarial drug treatment efficacy due to lack of funding. It is reassuring to note that the ACTs (AL and DHAPQ) were highly efficacious for the treatment of recurrent falciparum malaria. More importantly there were no patients with any persisting parasitaemia at day 3, indicating that delayed parasite clearance, a phenotype observed in south East Asia has not emerged or reached this Ugandan site.

This is the first study assessing the appropriate regimen for rescue therapy after ACT treatment in children. It is apparent that in areas of intense transmission, the risk of recurrent infection after quinine treatment, the current recommended second line regimen for Uganda and many other African countries, was unacceptably high compared to DHAPQ, which was highly efficacious and operationally preferable to quinine because of a less intensive dosing schedule. Therefore, in such settings DHAPQ could be an ideal second line regimen. In areas of less intense transmission, where the risk of recurrent infections is low, either DHAPQ or AL could be preferred to quinine because of their shorter dosing schedule and better tolerability, hence probably a better compliance. Unfortunately, more than half of the national malaria control programmes in sub-Saharan Africa still recommend monotherapy with oral quinine as second line treatment. Further, in routine practice the use of oral quinine as a first line treatment seems to be widespread [10]. Such a practice is not consistent with WHO guidance and should be urgently addressed to limit the selection pressure for quinine which is still an option for treating severe malaria.

### Generalizability

This study was conducted in a high transmission area and we believe our findings can be generalisable to other settings in Africa with similar malaria transmission intensity

### Limitations

The desired sample size of 260 on which power calculations were based could not be attained, affecting the power of the study. In addition, the number of patients in the AL group was disproportionately small (n = 35), presumably because failure within 28 days of DHAPQ in the main study was rare.

Randomization was dependent on previous treatment, as a result different groups had different randomization processes. All doses of the study regimens were given under direct supervision hence patients who received quinine had a longer interaction with the health care system. This could have enhanced the likelihood of detection of recurrent infection in the quinine group when compared with the other groups. However, all parents were encouraged to bring their children to the clinic whenever they were unwell.

### Overall evidence

Results from this study suggest that quinine is efficacious for the treatment of recurrent malaria when prescribed and properly administered. However, the PCR adjusted analysis may not show a statistically significant difference between quinine and the ACTs because of the small sample size. Furthermore, quinine effectiveness may be affected by poor adherence to treatment, particularly because of the long duration of treatment and of its low tolerability. Use of an ACT alternative to the first line treatment is preferable to oral quinine monotherapy because it would be better tolerated and highly effective against recurrent *P.falciparum* malaria. Several highly efficacious ACTs are now available and can be used as second-line treatments.

## Supporting Information

Checklist S1 **Completed CONSORT checklist.**
(DOC)

Protocol S1 **Study protocol.**
(DOC)

File S1 **InGenius Gel Documentation and Analysis system Standard Operating Procedure (SOP).**
(DOC)

File S2 **Makerere Institutional Review Board/Ethics Committee approval document.**
(PDF)

File S3 **Uganda National Council of Science and Technology approval document.**
(PDF)
