# Supplementary material for: Efficacy of Quinine, Artemether-Lumefantrine and Dihydroartemisinin-Piperaquine as Rescue Treatment for Uncomplicated Malaria in Ugandan Children
Source: PLoS One. 2013 Jan 22;8(1):e53772. doi: 10.1371/journal.pone.0053772 (PMC3551967; doi:10.1371/journal.pone.0053772)
Supplement: Protocol S1 — Study protocol. (DOC) [file pone.0053772.s002.doc]

**Evaluation of the best approach to retreating recurrent malaria in Ugandan children.**

**Co-ordinating Investigator**

**Name: Professor Umberto D'Alessandro PhD**

**Institution: Prince Leopold Institute of Tropical Medicine,**

**Address: Nationalestraat 155, B-2000, Antwerp, Belgium**

**Phone Number: 0032 3 247 6354**

**Fax Number: 0032 3 247 63 59**

**Email: udalessandro@itg.be**

| **Protocol No.:** | 1 |
| --- | --- |
| **Date:** | January 29^th^ , 2007 |
| **Version:** | One |
| **Title:** | Evaluation of the best approach to retreating recurrent malaria in Ugandan children. |
| **Country :** | Uganda. |
| **Sites :** | Nagongera Health Centre, Tororo District. |
| **Study drugs** | Dihydroartemisinin+Piperaquine , Artemether+Lumefantrine, Quinine |
| **Sponsor:** | Prince Leopold Institute of Tropical Medicine, Antwerp, Belgium |
| **PI** | Prof. Umberto D’Alessandro |
| **Local PI** | Dr Ambrose Talisuna. |
| **Local co-PI** | Dr. Adoke Yeka |
| **Address:** | Prince Leopold Institute of Tropical Medicine, Nationalestraat 155, B-2000, Antwerp, Belgium |
| **Telephone:** | 0032 3 247 6354 |
| **Fax:** | 0032 3 247 63 59 |
| **Email:** | [udalessandro@itg.be](mailto:udalessandro@itg.be) |

**Uganda Malaria Surveillance Project (UMSP) investigators.**

**COORDINATING INVESTIGATOR:**

Name: Professor Umberto D'Alessandro PhD

Institution: Prince Leopold Institute of Tropical Medicine,

Address: Nationalestraat 155, B-2000, Antwerp, Belgium

Phone Number: 0032 3 247 6354

Fax Number: 0032 3 247 63 59

Email: [udalessandro@itg.be](mailto:udalessandro@itg.be)

**CO-INVESTIGATORS:**

Name: Dr. Ambrose O. Talisuna MBchB, Msc, PhD

Institution: Coordinator African Artekin CoArtem Multi-Country Malaria Trial

Adress: Po Box 7272 Kampala ,Uganda

Phone Number: 256. 712. 506 275; 256. 414. 345 887

email: atalisuna@kla1.afsat.com or atalisuna@yahoo.com

Name: Fred Wabwire Mangen, MBChB, DTM&H, MPH, PhD

Institution: Makerere University Institute of Public Health

Address: Institute of Public Health, P.O. Box 7072, Kampala, Uganda

Phone Number: 256-414-543872

Fax Number: 256-414-531807

Email: fwabwire@iph.ac.ug

Name: Moses Kamya, MBChB, M Med, MPH

Institution: Makerere University Medical School

Address: Department of Medicine, P.O. Box 7072, Kampala, Uganda

Phone Number: 256-414-541188

Fax Number: 256-414-540524

Email: [malaria@infocom.co.ug](mailto:malaria@infocom.co.ug)

Name: Adoke Yeka, MBChB, MPH

Institution: Uganda Malaria Surveillance Project

Address: UMSP, P.O. Box 7475, Kampala, Uganda

Phone Number: 256-414-530692

Fax Number: 256-414-524540

Email: yadoke@yahoo.com

Name: Hasifa Bukirwa , MBChB, MSc

Institution: Uganda Malaria Surveillance Project

Address: UMSP, P.O. Box 7475, Kampala, Uganda

Phone Number: 256-414-530692

Fax Number: 256-41-524540

Email: h_bukirwa@yahoo.ca

**CONSULTANTS.**

Name: Philip Rosenthal, MD

Institution: University of California, San Francisco

Address: UCSF, Box 0811, San Francisco, CA 94143

Phone Number: 415-206-8845

Fax Number: 415-648-8425

Email: [rosnthl@itsa.ucsf.edu](mailto:rosnthl@itsa.ucsf.edu)

Name: Grant Dorsey, MD, MPH, PhD

Institution: University of California, San Francisco

Address: UCSF, Box 0811, San Francisco, CA 94143

Phone Number: 415-206-8687

Fax Number: 415-648-8425

Email: [grantd@itsa.ucsf.edu](mailto:staedke@itsa.ucsf.edu)

Name: Sarah G. Staedke, MD, DTM&H

Institution: London School of Hygiene and Tropical Medicine/ UMSP

Address: UMSP, P.O. Box 7475, Kampala, Uganda

Phone Number: 256-772-604844

Fax Number: 256-414-540524

Email: [sstaedke@medsfgh.ucsf.edu](mailto:sstaedke@medsfgh.ucsf.edu)

**UMSP SUPPORT STRUCTURES.**

| **Name** | **Institution** | **Title** |
| --- | --- | --- |
| Samuel Nsobya, MSc | M U | Chief Laboratory Technologist |
| John B. Rwakimare, MBChB, MPH | Uganda MOH | Technical Advisor |
| Thomas Kato, MBChB, MPH | Uganda MOH | Technical Advisor |
| Lugemwa Myers, MBChB, MPH | Uganda MOH | Technical Advisor |
| Namagembe Allen | UMSP | Data Manager |
| Nelson Budaka | UMSP | Laboratory Technologist |
| Catherine Tugaineyo | UMSP | Administrator |
| Nuhu Kibampawo | UMSP | Driver |
| Balikagala Betty | UMSP | Medical Officer |
| Mudhanga Fred | UMSP | Medical Officer |
| Musimenta Grace | UMSP | Nurse |
| Kizito Fred | UMSP | Laboratory Technologist |
| Byaruhanga Oswald | UMSP | Laboratory Technologist |
| TBH (2) | UMSP | Home Visitor |
| Heidi Hopkins, MD, MPH | UCSF | Technical Advisor |
| Oluga Richard | UMSP | Accountant |

**SYNOPSIS**

**Title**

Evaluation of the best approach to retreating recurrent malaria in Ugandan children.

**Methodology**

This is a nested phase IV, randomized, single blinded, multi-arm clinical trial of rescue therapy among patients who develop recurrent malaria within 28 days of treatment with Artemisinin-based combination treatment (ACT) in the main study. Details of the main study are described in the corresponding protocol (EDCTP trial protocol EDCTP-NCT00393679). Briefly, patients initially diagnosed with uncomplicated malaria will be randomized to one of the three ACT, namely Artemether + Lumefantrine (AL), Chlorproguanil-Dapsone+Artesunate, (CDA), or Dihydroartemisinin+Piperaquine (DP) and actively followed for 28 days and then passively until 6 months after treatment. If, during the passive follow up, the patient experiences a second uncomplicated malaria episode, they will be treated with the same drug and actively followed up for 28 additional days.

This study shall evaluate the efficacy and safety of quinine and two ACT’s (AL and DP) for treatment of recurrent uncomplicated malaria. Patients who develop recurrent uncomplicated malaria after day 3 of active follow up of the main study shall be recruited into this study. The patients will be randomized to either quinine or another ACT regimen (AL to quinine or DP, CDA to quinine, AL or DP, DP to quinine or AL) and followed for 28 days to assess their response to therapy. At the end of this follow up, patients will enter the 6-month period of passive surveillance in the main study. Patients shall be recruited into this trial for only one active follow up. The study follow up is summarised in the figure below.

**Follow up chart for rescue therapy.**

| **Day** | **0** | **1** | **2** | **3** | **4** | **5** | **6** | **7** | **14** | **21** | **28** | **Any other day^1^** |
| --- | --- | --- | --- | --- | --- | --- | --- | --- | --- | --- | --- | --- |
| History (symptoms) | X | X | X | X |  |  |  | X | X | X | X | X |
| Informed consent | X |  |  |  |  |  |  |  |  |  |  |  |
| Examination (clinical) | X | X | X | X |  |  |  | X | X | X | X | X |
| Temperature | X | X | X | X |  |  |  | X | X | X | X | X |
| Blood film | X |  | X | X |  |  |  | X | X | X | X | X |
| Filter paper PCR | X |  | X | X |  |  |  | X | X | X | X | X |
| Adverse drug reactions | X | X | X | X |  |  |  | X | X | X | X | X |
| Concomitant medications | X | X | X | X |  |  |  | X | X | X | X | X |
| Haematology | X |  |  |  |  |  |  | X | X | X | X | X |
| Treatment | X | X | X | X^1^ | X^1^ | X^1^ | X^1^ |  |  |  |  |  |
| Assessment for AEs | X | X | X | X | X | X | X | X | X | X | X | X |

X = perform this task

X^1^ = quinine treatment administration.

**Study Duration**

Calendar of activity.

| **YEAR** | **1** | | | | **2** | | | | **3** | | | |
| --- | --- | --- | --- | --- | --- | --- | --- | --- | --- | --- | --- | --- |
| **QUARTERS** | **1** | **2** | **3** | **4** | **1** | **2** | **3** | **4** | **1** | **2** | **3** | **4** |
| Monitoring initiation visit |  |  |  |  |  |  |  |  |  |  |  |  |
| Recruitment of patients |  |  |  |  |  |  |  |  |  |  |  |  |
| Monitoring visits |  |  |  |  |  |  |  |  |  |  |  |  |
| Interim analysis |  |  |  |  |  |  |  |  |  |  |  |  |
| Genotyping blood samples |  |  |  |  |  |  |  |  |  |  |  |  |
| Data Analysis |  |  |  |  |  |  |  |  |  |  |  |  |
| Publications |  |  |  |  |  |  |  |  |  |  |  |  |

**Country**

Uganda.

**Study Center**

Nagongera health centre, Tororo district.

**Objectives**

The main objective is to assess the best approach to retreating patients with recurrent malaria within 28 days of initial therapy with ACT’s.

Specific objectives are the following:

1. To evaluate the efficacy of AL, DP and quinine for the treatment of recurrent uncomplicated *P. falciparum* malaria within 28 days of initial therapy with another ACT.
2. To evaluate the safety and tolerability of AL, DP and quinine for the treatment of recurrent uncomplicated *P. falciparum* malaria.
3. To evaluate the selection of *Plasmodium falciparum* pfmdr1 alleles following therapy with quinine, AL, and DP.

**Number of Subjects**

130 patients/arm (quinine vs AL/DP), 260 in total.

**Diagnosis and Main Inclusion Criteria**

Participants who develop recurrent uncomplicated malaria during active follow up in the main study will be recruited into this study and randomised to one of the study treatments if they fulfil the following criteria. 1) Have recurrent *plasmodium falciparum* parasitaemia of any level. 2) the parents or guardians have signed the informed consent and 3) Parents or guardians willingness and ability to comply with the study protocol for the duration of the trial.

They will be excluded if they have at least one of the following criteria:

| 1. Early treatment failure in the main study (Days 0 – 3) |
| --- |
| 1. Known hypersensitivity to the study drugs |
| 1. Severe malaria*. |
| 1. Danger signs: not able to drink or breast-feed, vomiting (> twice in 24hours), recent history of convulsions (>1 in 24h), unconscious state, unable to sit or stand; |

*WHO 2000: Severe falciparum malaria. *Trans. R. Soc. Trop. Med. Hyg.* **94**, 1-90.

Children infected with other malaria species and those with mixed malaria infections will be excluded from the study. Cases of severe and complicated malaria will be referred to hospital for treatment/treated with intravenous quinine and other supportive therapy.

***Study endpoints***

**Primary end points**

1. PCR unadjusted treatment failure (TF28U): all treatment failures detected during the active follow up, regardless of genotyping.
2. PCR adjusted treatment failure up to day 28 (TF28A): all early failures before day 4 plus the recurrent parasitaemias detected at day 4 or later and classified by genotyping as recrudescence.

**Secondary endpoints.**

- - Fever clearance time.
  - Asexual parasite clearance time.
  - Gametocytaemia (prevalence and density) at day 7, 14, 21 and 28 after treatment;
  - Hb changes day 28 or day of treatment failure;
  - Change in the frequency of plasmodial genetic polymorphisms (pfmdr1) as longitudinal markers of antimalarial drug resistance
  - Safety profiles including significant changes in relevant laboratory values.

##### Statistical analysis

Data analysis will be primarily performed using SPSS and STATA statistical software packages. Descriptive statistics will be used to summarize baseline characteristics of study patients. Efficacy and safety data will be evaluated using a modified intention-to-treat analysis and will only include patients who meet all selection criteria. Categorical variables will be compared between the treatment groups using odds ratio, chi-square tests or Fisher’s exact tests and continuous variables will be compared using t-tests or non-parametric tests. A p-value of < 0.05 will be considered statistically significant. Estimates of the risk of failure for all primary outcomes will be made using the Kaplan-Meier product limit formula. The number (and percentage) of patients experiencing each adverse event will be compared between the treatment groups. No formal statistical testing will be undertaken. An interim report will be prepared when approximately one-half of the total projected sample size has been recruited (130 patients).

***Institutions involved***

- Institute of Tropical Medicine, Antwerp, Belgium:
- Liverpool School of Tropical Medicine and Centre for Medical Statistics and Health Evaluation, University of Liverpool, UK
- East African Network for Monitoring Antimalarial Treatment (EANMAT).
- Uganda Malaria Surveillance Project (UMSP), Kampala, Uganda.

# 1. RATIONALE AND BACKGROUND

Malaria remains one of the great infectious killers in Africa. An estimated 300 to 500 million cases occur each year, causing 1.5 to 2.7 million deaths, primarily in children under the age of five [1]. The reduction of malaria-associated morbidity and mortality relies largely on chemotherapy. An effective malaria vaccine is not likely to be available soon. Preventive measures directed against mosquito vectors have had limited success and, where some success has been seen with bed nets [2]), they have not been used widely enough to have much impact on malaria incidence, morbidity or mortality [3]. Indeed, the reduction in transmission levels necessary to impact greatly on clinical malaria is likely not achievable in many areas of Africa [4, 5]. Considering these facts, for the foreseeable future, the major intervention available for the control of malaria (and a key Roll Back Malaria priority) remains the prompt treatment of symptomatic malaria with effective therapy. However, the success of this strategy has been greatly affected by the increasing resistance of malaria parasites to available drugs. Resistance to chloroquine (CQ), the most widely used antimalarial, came to Africa some years after it was first noted in South America and Asia, but it is now widespread and increasing. Resistance to other agents, most notably sulphadoxine pyremethamine (SP), appears to be increasing. It is anticipated that the use of ineffective agents will lead to an increased incidence of disease [6], increased progression of disease from uncomplicated to complicated forms [7], and increased resultant mortality [8, 9]. Choices of the best treatment for uncomplicated malaria in Africa have become increasingly complex. Use of combination antimalarial therapy, particularly newer regimens containing artemisinin-based compounds, has been widely advocated. [10] However, concerns regarding the cost and availability of ACT remain. [11]

Several African countries have already opted for ACT’s. In Uganda, AL has already been chosen as first-line treatment for uncomplicated malaria. The recommended second line treatment for uncomplicated malaria is quinine. Oral quinine is widely used as a second line treatment for uncomplicated malaria in Africa. DP is a new very promising ACT that could be used extensively in the future. Despite the global momentum behind ACT’s, data on appropriate rescue therapy is still lacking. The need to provide African Ministries of Health with reliable information on ACT safety, efficacy and effectiveness and the appropriate rescue therapy is obvious. Assessment of the efficacy, and safety of new artemisinin regimens, evaluation of the best approach to retreating patients with recurrent malaria following ACT therapy, and assessment of quinine efficacy are all important research needs that will be addressed in this study.

## 1.1 Drugs to be tested

**Quinine.**

The alkaloids from the bark of the cinchona tree still constitute the mainstay of the antimalarial pharmacopeia, as they have for over three centuries. Quinine is widely used for the treatment of severe malaria. It is effective against all species of malaria including chloroquine-resistant strains of *P. falciparum*. Quinine belongs to the aryl amino alcohol group of drugs. It is a cinchona alkaloid that has a rapid schizonticidal action on the intra-erythrocytic parasites and is also gametocidal for *P. vivax* and *P. malariae* but not for *P. falciparum*.

In Uganda quinine is the second line treatment for malaria. It is cheap, widely available and generally considered to be effective but is not popular due to the unwanted side effects. Quinine has a very short half life, therefore repeated dosing is required. In an efficacy study of quinine and artemisinin for uncomplicated malaria in Vietnam, recrudescence rates were 16% after 7 days of quinine monotherapy [12]. In studies conducted in Gabon, *Plasmodium falciparum in vitro* sensitivity to quinine was high and had not changed over the past decade [13]. Although quinine monotherapy shows high efficacy in the setting of clinical trials, it has considerable disadvantages mainly because of its poor tolerability and the prolonged treatment course. Poor adherence carries a high risk of treatment failure, particularly because quinine causes a syndrome of adverse effects known as cinchonism, including primarily tinnitus, nausea, and vertigo. Other reported side effects include high tone hearing impairment, dizziness, hypotension as well as headache and visual disturbances [14]. As a result of these side effects some studies have reported poor compliance to treatment, a randomized trial in Thailand recorded a 71% rate of adherence [15].

### Artemether –lumefantrine (AL)

This is a fixed-dose combination of artemether (a semi-synthetic artemisinin derivative) and lumefantrine (a slowly eliminated drug also referred to as benflumetol). The registered indications and branding for AL cover treatment of uncomplicated malaria caused by mono or mixed *Plasmodium* infections. The combination is expected to confer mutual protection against resistance and prevent recrudescence after artemether therapy. The components of this combination were originally studied and developed in China by the Academy of Military Medical Sciences (AMMS), Beijing and Kunming Pharmaceutical Factory (KPF), Kunming. The fixed combination has been registered in China since 1992 and has undergone further development when Novartis signed a collaborative agreement in 1994 with AMMS, KPF and CITITEC, the technology arm of the China International Trust and Investment Corporation (CITIC). Studies for the international registration started in 1995. AL was registered in Switzerland in 1999 and has since received marketing authorisation in several endemic and non-endemic countries. Recently, an agreement was reached between Novartis and WHO for AL to be made available to the public sector of developing countries at a preferential price. Few clinical trials, mostly with the four-dose regimen, have been conducted both with the original Chinese combination product and the subsequent product used for international registration. A Cochrane review [17] has, in 2003, identified eight randomized, controlled trials comparing AL with standard treatment for uncomplicated falciparum malaria (2117 participants). From the meta-analysis, it was concluded that the four-dose AL regimen was superior to CQ and equivalent to SP in areas of CQ resistance but inferior to mefloquine and mefloquine-artesunate in areas of multidrug resistance. The six-dose regimen was also equivalent to mefloquine-artesunate but was better tolerated [18, 19]. A collaborative project between Novartis and WHO has led to a more user-friendly packaging of the six-dose treatment, which is now being field tested. A paediatric formulation is also being developed and trials have been conducted for regulatory submission to extend the label to cover children who weigh less than 10 kg [20].

### Dihydroartemisinin-piperaquine (DHAPQ)

DHAPQ was developed in China and is registered in China and Cambodia. Non GMP DHAPQ has been evaluated extensively in clinical trials in Thailand, Vietnam, Cambodia and China [21], efficacy has been high and tolerability uniformly good in all trials in multidrug-resistant areas, where piperaquine-resistance was common after its extensive use for mass prophylaxis. In Africa, DHAPQ has been evaluated in Rwanda in children 6-59 months old with uncomplicated malaria. The treatment was highly efficacious (95% adequate clinical and parasitological response at day 28 after treatment) and well tolerated [16]. Initially, the co-formulation included primaquine and trimethoprim (CV8), which is still part of national policy in Vietnam. Piperaquine is an orally active bisquinoline discovered by Rhône-Poulenc in the early 1960s and developed for clinical use in China in 1973. Piperaquine is approximately equivalent to chloroquine against sensitive parasites, but is significantly more effective than chloroquine against resistant *P. falciparum.* Piperaquine replaced chloroquine as the recommended treatment for falciparum malaria in China in 1978. Overall, 194 140·kg of piperaquine phosphate, equivalent to 140 000·000 adult doses, were used for mass prophylaxis and treatment. Surveillance at the time found no adverse events other than rare cases of a rash. However, reports about emerging parasite resistance to piperaquine from southern China, an area of intensive use of piperaquine, are of concern. Dihydroartemisinin is the active metabolite of artesunate and artemether. It has equivalent clinical efficacy to the more widely used artesunate. A development programme has been agreed between Holleykin Pharmaceuticals and Guangzhou University (China), The University of Oxford, MMV, and Sigma-Tau Industrie Farmaceutiche Riunite SpA to support the international registration of the drug. A multi-centre phase III clinical trial comparing DHAPQ with AL in Africa and with mefloquine+artesunate in Asia is ongoing.

**1.2. Molecular markers of drug resistance.**

Polymorphisms in certain parasite genes (pfcrt, pfmdr1, dhfr, dhps) have been associated with treatment responses to aminoquinolines and antifolates, but the importance of different mutations remains incompletely understood. Additional polymorphisms may impact upon host responses, and therefore potentially on the incidence of malaria.

Chloroquine (CQ) resistance is associated with an amino acid change from lysine to threonine in codon 76 of the *P. falciparum* chloroquine resistance transporter gene (pfcrt) [22], and a mutation from asparagine to tyrosine in codon 86 of the multidrug resistant gene (pfmdr) [23-26]. Sulphadoxine Pyremethamine (SP) resistance is associated with mutations in the dihydrofolate reductase (pfdhfr) and dihydropteroate synthase (pfdhps) genes. Pyrimethamine is a selective, competitive inhibitor of dihydrofolate reductase and earlier in the folate pathway sulpha drugs inhibit dihydropteroate synthase [27, 28]. Several point mutations are connected to antifolate drug resistance. The quintuple mutation (triple pfdhfr: I51, R59, N108 and double pfdhps, G437, E540) is discussed as a relevant molecular marker of SP treatment failure [29, 30]. Atovaquone-proguanil is a relatively new antimalarial drug that inhibits mitochondrial electron transport. Point mutations in the cytb codon 268 are associated with resistance to this combination [31-33].

There are indices that pfmdr1 N86 is associated with resistance to lumefantrine that is widely used in combination with artemether [34], and also with decreased sensitivity to artemisinins. Previous studies have shown that the pfmdr1 86N allele is associated with lower in vitro susceptibility to lumefantrine, halofantrine, and mefloquine (35 - 37). The pfmdr1 1246D allele is associated with decreased sensitivity to mefloquine and halofantrine in two transfection studies (38, 39). Interestingly, these alleles have been associated with an increased sensitivity to chloroquine (CQ) and are generally considered the wild-type alleles (35, 38). In a recent antimalarial drug efficacy study in Tanzania (40), the prevalence of the pfmdr1 86N allele increased from 15% in pretreatment samples to 41% in post treatment new infections. In a similar study in Uganda (41), the prevalence of samples containing only the pfmdr1 86N allele increased significantly from 8% in pretreatment samples to 43% in samples from patients with new infections. The prevalence of samples containing only the pfmdr1 184F allele increased significantly from 4% in pretreatment samples to 14% in samples from patients with new infections. The prevalence of samples containing only the pfmdr1 1246D allele increased significantly from 12% in pretreatment samples to 42% in samples from patients with new infections. There was significant concordance between the pfmdr1 86N and 1246D alleles in both pretreatment and post treatment samples. None of samples assayed contained the pfmdr1 1042D allele. The selection of the three polymorphisms in the pfmdr1 gene following administration of AL were not associated with clinical treatment failure but are evidence for the ability of this drug combination to drive selection of parasites toward resistant phenotypes.

Uganda and several other African countries are currently changing to AL as the first-line treatment for malaria. It is important to monitor the efficacy of AL closely, especially in high-transmission areas where drug selection pressure may be the greatest. The possible role of the pfmdr1 86N, 184F, and 1246D alleles and pfmdr1 copy number as molecular markers of AL resistance needs further investigation. We will evaluate polymorphisms in parasite genes, including those noted above, to search for associations between genotypes and treatment responses.

# 2. TRIAL OBJECTIVES AND PURPOSE

The main objective is to compare the safety and efficacy of 2 artemisinin-based combinations Dihydroartemisinin-piperaquine (DP), and Artemether-lumefantrine (AL) and quinine for retreating patients with recurrent malaria within 28 days of initial therapy with another ACT. Safety will be determined by registering adverse events and grading, laboratory, and vital signs evaluations. Their incidence will be compared between the different study arms.

Specific objectives are the following:

1. To evaluate the efficacy of AL, DP and quinine for the treatment of recurrent uncomplicated *P. falciparum* malaria within 28 days of initial therapy with another ACT.
2. To evaluate the safety and tolerability of AL, DP and quinine for the treatment of recurrent uncomplicated *P. falciparum* malaria.
3. To evaluate the selection of *Plasmodium falciparum* pfmdr1 alleles following therapy with quinine, AL, and DP.

# 3. TRIAL DESIGN

## 3.1 Study Design

This is a nested phase IV, randomized, single blinded, multi-arm clinical trial of rescue therapy among patients who develop recurrent malaria within 28 days of treatment with Artemisinin-based combination treatment (ACT) in the main study. Details of the main study are described in the corresponding protocol (EDCTP trial protocol EDCTP-NCT00393679). Briefly, patients initially diagnosed with uncomplicated malaria will be randomized to one of the three ACT, namely Artemether + Lumefantrine (AL), Chlorproguanil-Dapsone+Artesunate, (CDA), or Dihydroartemisinin+Piperaquine (DP) and actively followed for 28 days and then passively until 6 months after treatment. If, during the passive follow up, the patient experiences a second uncomplicated malaria episode, they will be treated with the same drug and actively followed up for 28 additional days.

This study shall evaluate the efficacy and safety of quinine and two ACT’s (AL and DP) for treatment of recurrent uncomplicated malaria. Patients who develop recurrent uncomplicated malaria after day 3 of active follow up of the main study shall be recruited into this study. The patients will be randomized to either quinine or another ACT regimen (AL to quinine or DP, CDA to quinine, AL or DP, DP to quinine or AL) and followed for 28 days to assess their response to therapy. At the end of this follow up, patients will enter the 6-month period of passive surveillance in the main study. Once a patient has entered the second phase of the study s/he will not be eligible anymore, even if s/he present with a recurrent parasitaemia after a second treatment with an ACT.

The main justification for not performing a double-blind study is the requirement to use the double-dummy technique requiring placebos for 3 arms drugs, resulting into a treatment schedule which would be very laborious for the patient. In order to ensure concealment of treatment allocation and avoid other biases, the following requirements will be assured:

1. The randomization list will be generated prior to the beginning of the study. Treatment allocation will be concealed until the completion of the screening and the final recruitment of the patient (see section 3.10);
2. The interpretation of the PCR reading will be blinded/masked with regard to the treatment allocation of the patients (see section 3.9);
3. An independent Data Monitoring Board will review all efficacy and safety data (see section 13.2).

## 3.2 Primary Endpoint

1. PCR unadjusted treatment failure (TF28U): all treatment failures detected during the active follow up, regardless of genotyping.
2. PCR adjusted treatment failure up to day 28 (TF28A): all early failures before day 4 plus the recurrent parasitaemias detected at day 4 or later and classified by genotyping as recrudescence.

The TF is defined according to the WHO criteria (WHO 2006) as the sum of early* and late** treatment failures.

* Early Treatment Failure (ETF) (one of the following)

(i) Development of danger signs or severe malaria (see Appendix V) on Day 0, Day 1, Day 2 or Day 3, in the presence of parasitaemia,

(ii) Parasite density on Day 2 > Day 0 count, irrespective of axillary temperature,

(iii) Presence of parasitaemia on Day 3 with fever (axillary temperature ≥ 37.5°C),

(iv) Parasitaemia on Day 3 ≥ 25 % of count on Day 0.

** Late treatment failure (LTF)

LTF is divided in late clinical and late parasitological failure.

Late Clinical Failure (LCF):

(i) Development of danger signs or severe malaria after Day 3 in the presence of parasitaemia, (See Appendix V for the criteria of severe malaria/danger signs).

(ii) Presence of parasitaemia and fever on any day from Day 4 to Day 28, without having previously meet the criteria of ETF.

Late Parasitological Failure (LPF):

Reappearance of parasitaemia after day 3 in the absence of fever (axillary temperature <37.5°C) without having previously meet the criteria of ETF or LCF.

The adequate clinical and parasitological response (ACPR) is 1-TF (28 unadjusted or adjusted). It is defined as absence of parasitaemia at the end of the follow up period (day 28), irrespective of axillary temperature without previously meeting any of the criteria of early and late treatment failure. In the adjusted estimates, patients with late asexual parasite reappearance (with or without fever) will be considered ACPR if the PCR analysis shows a new infection rather than a recrudescence.

## 3.3 Secondary Endpoints

The secondary endpoints will be:

- - Fever clearance time (FCT): Fever clearance time will be defined as the time (in days) from the time of randomization to the first two consecutive measurements on 2 different days of axillary temperature below 37.5°C.
  - Asexual parasite clearance time (PCT): Asexual parasite clearance time will be defined as the time (in days) from time of randomization to 2 consecutive negative blood slides (collected at different days). The time to the event will be taken as the time to the first negative slide.
  - Gametocytaemia (prevalence and density) at day 7, 14, 21 and 28 after treatment;
  - Hb changes day0, 3, 7, 14 and 28.
  - Change in the prevalence of pfmdr1 alleles (86N, 184F, 1042D, 1246D) associated with drug resistance from day 0 to the day of recurrent parasitaemia.
  - Safety profiles: Subjects will be monitored for 28 days for possible development of adverse events. All adverse events will be recorded on the specific form in the CRF. Vital signs, and haematology will be monitored and changes in relevant laboratory parameters will be assessed.

**3.4 PCR analysis**

**3.4.1. Genotyping**.

Genotyping of the recurrent infection will be done by characterizing MSP1, MSP2 and 4 micro satellites single-copy genes in the *Plasmodium falciparum* genome. PCR-amplification of DNA from a single parasite clone results in a single amplification product. For the three genes, each PCR-amplification product of a different size is considered to originate from a different clone of *Plasmodium falciparum* and reflects a different genotype. For the samples collected from the same patient at day 0 and day of recurrent parasitaemia, the length polymorphism of MSP1, MSP2 and 4 micro satellites will be determined, i.e. the number of bands in each PCR reaction and their respective size. Results will be interpreted as follows:

**3.4.2. Molecular markers:**

Alleles for molecular markers shall be identified using nested PCR and restriction fragment length polymorphism methods, and copy number shall be assessed by TaqMan quantitative PCR as previously described (35, 37). Single (3D7) - and three-copy (W2mef) standards shall be used as positive controls. Reactions shall be done in quadruplicate and repeated for a change in cycle threshold standard error >0.3 or a copy number between 1.3 and 1.6. A copy number <1.5 shall be considered single copy, and one ≥1.5 shall be considered multiple copy (37).

**3.4.3. Interpretation of PCR results**

***Recrudescence***: For each marker (MSP1, MSP2 and 4 micro satellites), at least one identical length polymorphism is found in the sample collected at day 0 and day of recurrent parasitaemia.

***New infection***: For at least one marker, length polymorphism is different between the sample collected at day 0 and that at day of recurrent parasitaemia.

***Indeterminate***: Samples that failed to produce a result due to an inability to amplify DNA at day 0 and/or day of recurrent parasitaemia.

**3.5 *Sample size***

The samples size is determined by the primary study. There will be a total of 510 children with uncomplicated malaria recruited into the primary study (170 per arm). We estimate that at least 51% of these patients will have an LCF or LPF response between days 4 and 28 based on previous studies at the site [42]. Thus we will end up with an estimated 260 patients randomized to Quinine vs. AL or DP (130 on quinine and 130 on AL or DP). We further assume that 50% of patients treated with AL or DP will be parasitological treatment failures unadjusted by genotyping (ETF, LCF or LPF). Assuming 90% of patients will complete follow-up, we will have 80% power (alpha level = 0.05) to detect a significant difference between the two treatment groups (quinine vs. ACT) if the risk of failure in the quinine group is about 65-70%.

##### 3.6 *Statistical analysis*

Data analysis will be primarily performed using SPSS and STATA statistical software packages. Descriptive statistics will be used to summarize baseline characteristics of study patients. Efficacy and safety data will be evaluated using a modified intention-to-treat analysis and will only include patients who meet all selection criteria. Categorical variables will be compared between the treatment groups using odds ratio, chi-square tests or Fisher’s exact tests and continuous variables will be compared using t-tests or non-parametric tests. A p-value of < 0.05 will be considered statistically significant. Estimates of the risk of failure for all primary outcomes will be made using the Kaplan-Meier product limit formula. Patients excluded after enrollment will be censored at the time of their last assessment. Additionally, for genotyping adjusted outcomes, patients with recurrent malaria or recurrent parasitemia due to new infections will be censored. Stratified analysis shall be done for outcomes in patients with recurrent symptomatic (ETF, LCF) and recurrent asymptomatic (LPF) malaria. The number (and percentage) of patients experiencing each adverse event will be compared between the treatment groups. No formal statistical testing will be undertaken. An interim report will be prepared when approximately one-half of the total projected sample size has been recruited (130 patients). The interim report will contain information on study progress and data quality (including subject recruitment, patient follow-up, and protocol adherence), safety data (serious adverse events). In addition, the clinical monitor will be asked to review any serious adverse events identified during the study.

**3.7 *Duration of patient follow up***

Each patient will be followed up actively after recruitment for 28 days.

## 3.8 Follow-up Chart

All children recruited in the main study (EDCTP trial protocol EDCTP-NCT00393679) who present with recurrent uncomplicated malaria and meet the recruitment criteria will be included in the study. The following steps will be taken:

1. A Case Report Form consisting of demographic and physical/clinical information will be completed;
2. Body weight and axillary temperature will be measured (the latter by using a digital electronic thermometer) and the results will be recorded on the Case Report Form. If the initial temperature value is less than 36°C the measurement will be repeated.
3. A thick blood smear will be obtained for measuring parasite density and a thin blood film for malaria species determination (the sample collected for screening can be used).
4. A blood sample will be collected on Whatman filter paper number 3MM for subsequent genotyping of the infective parasite strains.

All study medications for patients randomised to ACT’s shall be administered under direct observation. Study medications for patients randomised to quinine shall be administered under direct observation on the first 3 days (9 doses). For the remaining quinine doses, the two doses for the day shall be administered in the clinic under direct supervision by the study nurse and the third dose for the day shall be administered by the parents or guardians at home. Patients will have the option to be admitted for observation and study drug administration or to commute from home. Parents/guardians will be encouraged to return to the clinic for follow up assessments on days 3, 7, 14, 21, 28 and on any unscheduled day if the child is not well.

Patients will be assessed as summarized in the following flow-chart.

**Follow up chart.**

| **Day** | **0** | **1** | **2** | **3** | **4** | **5** | **6** | **7** | **14** | **21** | **28** | **Any other day^1^** |
| --- | --- | --- | --- | --- | --- | --- | --- | --- | --- | --- | --- | --- |
| History (symptoms) | X | X | X | X |  |  |  | X | X | X | X | X |
| Informed consent | X |  |  |  |  |  |  |  |  |  |  |  |
| Examination (clinical) | X | X | X | X |  |  |  | X | X | X | X | X |
| Temperature | X | X | X | X |  |  |  | X | X | X | X | X |
| Blood film | X |  | X | X |  |  |  | X | X | X | X | X |
| Filter paper PCR | X |  | X | X |  |  |  | X | X | X | X | X |
| Adverse drug reactions | X | X | X | X |  |  |  | X | X | X | X | X |
| Concomitant medications | X | X | X | X |  |  |  | X | X | X | X | X |
| Haematology | X |  |  |  |  |  |  | X | X | X | X | X |
| Treatment | X | X | X | X^1^ | X^1^ | X^1^ | X^1^ | X^1^ |  |  |  |  |
| Assessment for AEs | X | X | X | X | X | X | X | X | X | X | X | X |

X = perform this task

X^1^ = quinine treatment administration.

## 3.9. Selection of the Patients

### Inclusion criteria

In order to be eligible, patients should satisfy the following inclusion criteria:

1. Males and Females aged between 1- 6 years inclusive.
2. Recurrent *Plasmodium falciparum* infection.
3. Parents’ or guardians’ willingness and ability to comply with the study protocol for the duration of the trial.

### Exclusion criteria

Patients with any of the following criteria will not be admitted to the study:

1. Known hypersensitivity to the study drugs.
2. Severe malaria.
3. Danger signs: not able to drink or breast-feed, vomiting (> twice in 24hours), recent history of convulsions (>1 in 24h), unconscious state, unable to sit or stand.
4. Early treatment failure in the main study.

WHO recommends treatment of all cases with LPF since majority of them become symptomatic during prolonged follow up.

## 3.10 Study Procedures

The critical steps for the study period are described in Appendix VII.

**Day 0: screening visit/ administration of the study medication**

1. Physical and Clinical Examination

A general physical examination will be performed (see Appendix I).

A clinical examination will be performed (see Appendix I): symptoms and axillary temperature (electronic thermometer).

1. Weight

Weight will be measured.

1. Blood Slide

A thick and thin blood smear will be obtained from the subject to verify the presence of *P. falciparum* and to calculate the parasite density. Thick and thin blood films will be prepared, dried and stained with Giemsa stain according to standard operating procedures.

Parasite density will be calculated by counting the number of asexual parasites per 200 leukocytes in the thick blood film, based on an assumed WBC of 8,000 /µl by light microscopy at 1000 X magnification. One hundred high-powered fields (HPF) will be examined (independent of presence or absence of asexual parasite stages). The parasite density per microlitre will be calculated using the following formula:

Parasite density / µl = Number of parasites counted x 8,000

Number of leukocytes counted

1. PCR

A blood sample will be collected on filter paper (Whatmann 3MM) at day 0 before treatment and from day 4 onwards, every time a blood slide is done. Samples from patients classified as late treatment failure will be subsequently used for parasite genotyping.

1. Laboratory Tests

Blood haemoglobin, and other tests that the clinician may request for.

1. Administration of the Study Drugs
2. Adverse Events Report

All adverse events will be recorded. See section 7.1.4 for the details on the information collected.

10 Concomitant Medications

Any medications taken by the study subject will be recorded in the CRF.

**Day 1 and 2: *Open Label Treatment Period***

1. Physical and Clinical Examination

A general physical examination and a clinical examination will be performed: symptoms, axillary temperature (electronic thermometer).

1. Blood Slide

A thick blood smear will be obtained on Day 2 to check for presence and density of asexual and sexual stages of *P*. *falciparum*.

1. Concomitant Pharmacological Treatments

Concomitant medications being taken by the patients will be recorded. For a list of allowed and disallowed medications, see section 4.

1. Adverse Events Report

All adverse events will be recorded. See section 7.1.4 for the details on the information collected

1. Administration of the Study Drugs

**Day 3: *Open Label Treatment Period***

1. Physical and Clinical Examination

A general physical examination and a clinical examination will be performed: symptoms, axillary temperature (electronic thermometer).

1. Blood Slide

A thick blood smear will be obtained to determine the presence and the density of asexual and sexual stages of *P*. *falciparum.*

1. Concomitant Pharmacological Treatments

Concomitant medications being taken by the patients will be recorded. For a list of allowed and disallowed medications, see section 4.

1. Adverse Events Report

All adverse events will be recorded. See section 7.1.4 for the details on the information collected

***Day 4-6: Quinine group Treatment Period***

1. Administration of the Study Drugs for quinine .

**Day 7, 14 and 21: *Open Label Treatment Period***

As at Day 3. In addition, the following information will be collected:

1. Medical History

Symptoms and other relevant episodes between visits will be recorded.

1. PCR

A blood sample will be collected on filter paper (Whatmann 3MM) for later genotyping.

**Day 28: *Open Label Treatment Period***

As Day 7.

1. PCR

A blood sample will be collected on filter paper (Whatmann 3MM) for later genotyping.

1. Laboratory Tests

Blood haemoglobin, and other tests that the clinician may request for.

**Unscheduled visits throughout follow up**

During this visits, the same procedures as day 21 will be applied. Haemoglobin will be measured if the patient is classified as treatment failure.

The PCR readings will be centralised and masked to the treatment allocation of study subjects. It will be done by personnel different from the treating physician/investigator. In addition, a centralized and independent double-check of at least 10% of blood slides and filter paper blood samples (PCR) will be carried-out. The percentages of slides and films to be reviewed and the corresponding statistical justifications will be specified in the Statistical Analysis Plan.

***3.11 Randomisation and treatment allocation***

Patients will be randomly assigned to quinine or another ACT. The randomization list will be computer generated by a member of the project who will not be directly involved in the conduct of the study. Only the study nurse will have access to the sealed treatment randomization list. At the study site, treatment allocation and administration of medications will be performed by the study nurse.

***3.12 Treatment administration***

The study treatment will be administered under the direct supervision of a study nurse. The patient will have the option of being admitted for the first 3 days of treatment administration or going home and returning to the clinic for the remaining doses. For patients who choose to go home between doses, a home visitor will collect information on where they live. If a patient fails to return to the clinic in a timely manner for their daily dose of study drug, they will be visited at home and brought to the clinic the same day. If patients miss any dose of study drugs, they will not be excluded from the study. The study nurse will record the date and time study drugs are administered. From day 3 to 6 the remaining quinine doses shall be administered. The two doses for the day shall be administered in the clinic under direct supervision and the third dose for the day shall be administered by the parents/guardians at home. Study drugs given to young children will be crushed, mixed with water, and administered as a slurry. Study drugs administered to older children will be given as tablets or fractions of tablets to be taken orally with a glass of water. The correct number of tablets will be determined using the weight dosing chart. After drug administration, patients will be kept for thirty minutes in the clinic. A dose will be repeated in full if vomiting occurs within 30 minutes of administration. This event will be documented in the case record form (CRF). If vomiting persists beyond two additional doses, the patient will be withdrawn from the study and treatment changed.

# 4. CONCOMITANT THERAPIES

## 4.1 Disallowed Concomitant Drug Therapies during the active follow ups

Any antimalarial, or antibiotic with antimalarial activity (erythromycin or other macrolides, co-trimoxazole or other sulfonamides, any tetracycline including doxycycline, and quinolones, clindamycin). Intake of these drugs leads to withdrawal of the patient from active follow up.

## 4.2 Allowed Concomitant Drug Therapies

During the trial, patients can be prescribed drugs e.g. paracetamol, and antibiotics with no known antimalarial activity (penicillins, cephalosporins). The dose, route, time and duration of any concomitant medical treatment will recorded in the CRF.

## 4.3 Special Conditions

Parents or guardians will be discouraged from obtaining drugs from any other source such as private pharmacies, markets or clinics. Parents/guardians will be encouraged to bring their children to the study clinic if their child is unwell or if they are worried about their child's health.

## 4.4 Rescue Treatments

All patients who develop severe/complicated malaria during active follow-up will be treated with parenteral quinine. Patients randomised to ACT who fail therapy will be treated with quinine 10 mg/kg orally three times a day for 7 days. Patients who fail quinine therapy shall be treated with Artesunate (2mg/kg once a day) + clindamycin (10mg/kg twice a day) for 7 days.

# 5. PATIENT WITHDRAWAL CRITERIA

Patients will be excluded from further assessment if there is withdrawal of informed consent. Severe adverse events related to the study drug are also a reason for withdrawal from the study.

# 6. PROTOCOL VIOLATIONS

A protocol violation occurs when an event happens that does not allow for accurate interpretation of response to treatment. Protocol violations will be defined in the statistical analysis plan.

# 7. SAFETY VARIABLES

Safety and tolerability of the treatments will be evaluated by recording Adverse Events (AEs) and grading, laboratory, and vital signs evaluations.

## 7.1 Adverse Events

At each visit, the Investigator will ascertain the occurrence of any adverse events since the last visit. Any event must be recorded on the CRF.

### 7.1.1 *Definition of an adverse event*

An AE is any untoward medical occurrence in a patient or clinical investigation subject administered a pharmaceutical product and which does not necessarily have a causal relationship with this treatment.

An AE can therefore be any unfavourable and unintended sign (that could include a clinically significant abnormal laboratory finding), symptom or disease temporally associated with the use of a medicinal product, whether or not considered related to the medicinal product.

### 7.1.2 *Severity, relationship of event to study drug, and outcome*

The severity of a clinical adverse event is to be scored according to the following scale:

1 Mild Awareness of sign or symptom, but easily tolerated

2 Moderate Discomfort enough to cause interference with usual activity

3 Severe Incapacitating with inability to work or perform usual activity

4 Life-threatening Patients at risk of death at the time of the event

The relationship of an adverse event to study drug is to be assessed according to the following definitions:

1 Definitely unrelated

Should be reserved for those events which occur prior to test drug administration (e.g., washout or single-blind placebo) or for those events which cannot be even remotely related to study participation (e.g. injury caused by a third party).

2 Unlikely

There is no reasonable temporal association between the study drug and the suspected event and the event could have been produced by the subject's clinical state or other modes of therapy administered to the subject.

3 Possible

The suspected adverse event may or may not follow a reasonable temporal sequence from study drug administration but seems to be the type of reaction that cannot be dismissed as unlikely. The event could have been produced or mimicked by the subject's clinical state or by other modes of therapy concomitantly administered to the subject.

4 Probable

The suspected adverse event follows a reasonable temporal sequence from study drug administration, abates upon discontinuation of the drug, and cannot be reasonably explained by the known characteristics of the subject's clinical state.

5 Definitely related

Should be reserved for those events which have no uncertainty in their relationship to test drug administration: this means that a rechallenge was positive.

The outcome of each AE must be assessed according to the following classification:

| - completely recovered : | The patient has fully recovered with no observable residual effects |
| --- | --- |
| - not yet completely recovered : | Improvement in the patient’s condition has occurred, but the patient still has some residual effects |
| - deterioration : | The patient’s overall condition has worsened |
| - permanent damage : | The AE has resulted in a permanent impairment |
| - death : | The patient died due to the AE |
| - ongoing : | The AE has not resolved and remains the same as at onset |
| - unknown : | The outcome of the AE is not known because the patient did not return for follow-up (lost to follow-up) |

### 7.1.3 *Definition of a serious adverse event*

A serious adverse event (experience) (SAE) or reaction is any untoward medical occurrence that at any dose fulfils at least one of the following criteria:

- results in death;
- is life-threatening;
- requires hospitalization (other than for drug administration) or prolongation of existing hospitalization;
- results in persistent or significant disability/incapacity;
- requires a specific medical or surgical intervention to prevent one of the outcomes listed above;

**All serious adverse events, whether or not deemed drug-related, or expected, must be reported immediately or within 24 hours (one working day), using the Serious Adverse Event Notification Form, by telefax or email to :**

Uganda Malaria Surveillance Project Study Coordinator

Mulago Hospital Complex

Room C 10 Anatomy Building

Tel: 041.530.692

Fax: 041. 540.524

Email: yadoke@yahoo.com

Fax should state “Urgent Serious Adverse Event” on cover page.

All other AEs not fulfilling the criteria of immediate reporting must be recorded on the Case Report Form. This AE information will be collected on a regular basis during the clinical trial. Considering that the study drugs have already been tested in several hundreds of patients and that the study is not a double-blinded one, a Local Safety Monitor is not needed.

### 7.1.4 *Reporting of adverse events*

For all adverse events identified, an adverse event report form will be completed.

For each possible adverse event identified and considered as **serious**, a serious adverse event notification form will be completed.

The following information will be recorded for all adverse events:

1. Study randomization number
2. Description of event
3. Date of event onset
4. Date event reported
5. Severity of the event
6. Possible relationship of the event to study medication
7. Is the event serious?
8. Initials of the person reporting the event
9. Was the event episodic or intermittent in nature?
10. Outcome of adverse event
11. Action taken
12. Date event resolved.

A severity grading scale, based on toxicity grading scales developed by the WHO and the National Institutes of Health, Division of Microbiology and Infectious Diseases, will be used to grade severity of all symptoms, physical exam findings, and haemoglobin results (see Appendix I). Any new event, or an event present at baseline that is increasing in severity, will be considered as an adverse event.

**7.1.5** *Length of follow-up for adverse events*

**AEs presenting during the active follow-up period:** A patient still experiencing an AE at the end of the active follow up, i.e. at day 28 will be managed as follows:

- If the AE has been detected and reported before the last visit and
  - it is mild (Grade 1), the patient will be managed according to good medical practice and the active follow up will be stopped. The end date for the AE will be recorded as Day 28.
  - If its grade is more than 1, the patient will be followed until the AE resolves, improves, or stabilizes.
- If the AE is new, the AE will be reported and the patient will be followed until the AE resolves, improves, or stabilizes.

**For patients classified as clinical treatment failures (ETF/LCF/LPF):**

Formal study follow-up ends when a patient is classified as a treatment failure (ETF or LTF/LPF), and patients should be treated and managed according to good medical practice. Additional follow-up for AEs in patients classified as treatment failures, is not typically indicated, unless the AE is serious, or is felt to be probably or definitely related to the study medications..

**For patients with Serious Adverse Events (SAEs):**

Any patient who experiences a Serious Adverse Event should be followed until the SAE resolves or improves (< grade 1). Although formal study follow-up is typically terminated when a patient is classified as a treatment failure, any patient with severe malaria / danger signs should be followed up to ensure their SAE has resolved / improved.

## 7.2 Laboratory Evaluations

Blood samples will be properly labelled with patients' initials, study number, the study day and the date the sample is taken. Haematology assessments will be performed locally at sites.

All laboratory results will be reported in Standard International Units or in conventional units.

Blood samples collected on filter paper for PCR genotyping will be analysed at the Institute of Tropical Medicine, Antwerp, Belgium. Sample will be collected according to standard operating procedures.

# 8. CASE REPORT FORM (CRF)

Presentation of the CRF

The CRF to be used for the study consists of pages headed with the study code and other relevant information. It is composed of an introductive part for the selection and inclusion of patients in the study and special forms for the different evaluation times; at the end of the CRF are the forms for registration of possible adverse events. Each page includes a header containing information for the identification on the study.

How to use the CRF

The CRF will be filled out using a ballpoint pen with black or blue ink. All requested information shall be entered on the CRFs. If an item is not available or is not applicable, this fact should be indicated; there should be no blank spaces. A correction should be made by striking through the incorrect entry with a single line and by entering the correct information adjacent to it. The correction must be initialled, dated and explained if necessary by the Investigator or by a qualified individual specifically designated by the Investigator. Each completed Case Report Form must be reviewed, signed and dated by the Investigator or by a qualified individual specifically designated by the Investigator.

# 9. MONITORING AND QUALITY ASSURANCE

The task of the Monitor is to verify the best conduct of the study through frequent contacts by phone and in person with the Principal Investigator and site staff, in accordance with the Standard Operating Procedures and Good Clinical Practice, with the purposes of facilitating the work and attaining the objectives of the study. These visits will enable the Monitor to maintain current, personal knowledge of the study through review of the records, comparison with source documents, observation and discussion of the conduct of the study with the Investigator. The site will be visited 3 times during the conduct of the trial. The monitor will carry out 30% source data verification.

The investigator shall maintain source documents for each patient in the study, consisting in case and visit notes (hospital or clinic medical records) containing demographic and medical information, laboratory data, and the results of any other tests or assessments. All information on the CRF shall be traceable to these source documents in the patient’s file. The investigator shall keep the original informed consent form signed by the patient. The investigator shall give the monitor access to all relevant source documents to confirm their consistency with the CRF entries.

The Investigator agrees to conduct the present study in full agreement with the principles of the “Declaration of Helsinki” and subsequent relevant amendments (see Appendix III).

# 10. DATA MANAGEMENT

Clinical data, as requested in the protocol, will be collected and recorded on appropriate paper Case Report Forms (CRF’s). Laboratory data, as requested in the protocol will be registered at the laboratory and recorded onto the CRF. All data will then be processed from the CRF’s into an electronic database. During the conduct of the study, data will be verified and reviewed to produce and maintain high quality data. All unresolved issues will be queried and resolved before locking the database. Data transfer and handling is done with appropriate security measures and with regard to rights, safety and well-being of trial subjects.

A report on data management process will be produced. The report will include

- A full field listing and description of the file structure of the electronic data
- Reference ranges and units for laboratory data
- A list a brief description of all programs run on the data
- Level of errors found at each stage of checking the data
- General comments on data quality and significant problems encountered with the data
- A detailed list of any unresolved data queries
- A statement of any queries/errors which have not been corrected on the database
- A statement of the storage location of the electronic database

The statistician will review the database prior to finalisation and report on any problem encountered during the analysis.

# 11. INVESTIGATOR RESPONSIBILITY

The term "Investigator" as used in this protocol and on the CRFs refers to the Principal Investigator or a member of the staff that the Investigator designates to perform a certain duty under this protocol. The Investigator is ultimately responsible for the conduct of all aspects of the study. For all other relevant Investigator responsibilities see “CPMP/ICH/135/95 Topic E6 - Guideline for Good Clinical Practice”, Chapter 4.

# 12. ADMINISTRATIVE PROCEDURES

## 12.1 Regulatory Authorities and Ethical Review Committee

This study will be submitted for approval by the Makerere University Review and Ethics Committee and the Uganda National Council of Science and Tehnology before patients can be enrolled.

## 12.2 Informed Consent

All interviews will be conducted in the native language of the patients by the study personnel. Consent forms in the local language will be provided to the parents or guardians for their review (see Appendix IV). The parents or guardians will be asked to sign (or thumb-print whenever the parents/guardians are illiterate) consent to participate in a research study. The informed consent will describe the purpose of the study, the procedures to be followed, and the risks and benefits of participation. If a parent or guardian is unable to read or write, a signature from a witness to the informed consent discussion will be obtained. Parents or guardians will be informed that participation in the study is completely voluntary and that they may withdraw their child from the study at any time without any negative consequences.

## 12.3 Confidentiality and Publication of Results

All study documents are provided by the investigators and his/her appointed staff. None of this material may be disclosed to any part not directly involved in the study without written permission from the Investigator.

## 12.4 Protocol Amendments

Clinical protocol amendments are alterations to a legal document (the clinical protocol) and have the same legal status and must pass through the appropriate steps before being implemented. In general, any change must be approved by the IEC prior to be effective. Administrative changes need only notification to the IEC without approval. Any subsequent amendments shall be made on separate sheets and must pass through the approval process.

# 13. DATA SAFETY AND MONITORING BOARD

A Data and Safety Monitoring Board (DSMB) will be established for the purpose of providing an independent advice on safety of the treatments tested. The DSMB will be composed of three members with expertise in malaria, biostatistics and other appropriate disciplines. The DSMB will function as an independent body: it will regularly review interim analysis reports and will be informed by the sponsor on any serious adverse event occurring during the trials. The DSMB will be able to make decisions on whether the trial, or arms of the trial, needs to be stopped. A Trial Monitor will inspect the site and trial documents/records.

The members of the DMB will be identified prior to enrolling the first patient.

# 14. ETHICAL ISSUES

AL has been successfully used for the treatment of falciparum malaria in Phase III studies. AL is widely used to treat uncomplicated malaria in Africa. DHAPQ has been used for several years in Asia, including safety studies in children. Recent trials in Rwanda and Peru (initiated and followed up by the ITM, Belgium) do not show any specific safety problem [18]. The combination is well tolerated. In Uganda quinine is the second line treatment for malaria. It is cheap, widely available and generally considered to be effective

The study will be presented for ethical clearance to the Makerere University Review and Ethics Committee and the Uganda National Council of Science and Technology. Prior to the start of the project, the study will be explained to the communities involved. Written informed consent will be obtained from the guardians for all children before entering the study.

# 15. REFERENCES

1. Breman, J.G., (2001) The ears of the hippopotamus: manifestations, determinants, and estimates of the malaria burden. Am J Trop Med Hyg 64(1-2 Suppl): 1-11.
2. D'Alessandro, U., B.O. Olaleye, W. McGuire, P. Langerock, S. Bennett, M.K. Aikins, M.C. Thomson, M.K. Cham, B.A. Cham, and B.M. Greenwood, (1995) Mortality and morbidity from malaria in Gambian children after introduction of an impregnated bednet programme. Lancet 345(8948): 479-83.
3. Alnwick, D., (2000) Roll back malaria--what are the prospects? Bull World Health Organ 78(12): .
4. Rogier, C., A. Tall, N. Diagne, D. Fontenille, A. Spiegel, and J.F. Trape, (1999) Plasmodium falciparum clinical malaria: lessons from longitudinal studies in Senegal. Parassitologia 41(1-3): 255-9.
5. Marsh, K. and R.W. Snow, (1999) Malaria transmission and morbidity. Parassitologia 41(1-3): 241-6.
6. Nosten, F., M. van Vugt, R. Price, C. Luxemburger, K.L. Thway, A. Brockman, R. McGready, F. ter Kuile, S. Looareesuwan, and N.J. White, (2000) Effects of artesunate-mefloquine combination on incidence of Plasmodium falciparum malaria and mefloquine resistance in western Thailand: a prospective study. Lancet 356(9226): 297-302.
7. White, N., (1999) Antimalarial drug resistance and mortality in falciparum malaria. Trop Med Int Health 4(7): 469-70.
8. Trape, J.F., G. Pison, M.P. Preziosi, C. Enel, A. Desgrees du Lou, V. Delaunay, B. Samb, E. Lagarde, J.F. Molez, and F. Simondon, (1998) Impact of chloroquine resistance on malaria mortality. C R Acad Sci III 321(8): 689-97.
9. Trape, J.F., (2001) The public health impact of chloroquine resistance in Africa. Am J Trop Med Hyg 64 (suppl): 12-17.
10. Kremsner PG, Krishna S (2004) Antimalarial combinations. Lancet 364: 285-294.
11. Snow RW, Eckert E, Teklehaimanot A (2003) Estimating the needs for artesunate-based combination therapy for malaria case-management in Africa. Trends Parasitol 19: 363-369.
12. Peter J. Deveis, Nguyen Ngoc Bich, Huynh Van Thien, Le Ngoc hung, Trinh Kim Anh, Piet A. Kager, and Siem H. Heisterkamp. Combination of artemisinin and quinine for uncomplicated falciparum malaria: Efficacy and pharmacodynamics. Antimicrobial agent and chemotherapy, May 2000, p. 1302-1308.
13. Ramharter M, Wernsdorfer WH, Kremsner PG, 2004. In vitro activity of quinolines against Plasmodium falciparum in Gabon. Acta Trop 90: 55–60.
14. Ayôla A. Adegnika*, Lutz Ph. Breitling, Selidji T. Agnandji, Sanders K. Chai, Daniela Schütte, Sunny Oyakhirome, Norbert G. Schwarz, Martin P. Grobusch, Michel A. Missinou, Michael Ramharter, Saadou Issifou, And Peter G. Kremsner. Effectiveness Of Quinine Monotherapy For The Treatment Of Plasmodium Falciparum Infection In Pregnant Women In Lambaréné, Gabon. Am. J. Trop. Med. Hyg., 73(2), 2005, pp. 263-266.
15. C. Karema, CI. Fanello, C. Van Overmeir, JC. Dujardin, JP. Van geertruyden, W. Van Doren, D. Ngamije, U. D’Alessandro. Safety and efficacy of dihydroartemisinin-piperaquine (Artekin®) for the treatment of uncomplicated *Plasmodium falciparum* malaria in Rwandan children. *Trans. R. Soc. Trop. Med. Hyg.* (in press).
16. Omari, A. A., Preston, C. and Garner, P.Artemether-lumefantrine for treating uncomplicated falciparum malaria.*Cochrane Database Syst. Rev. 2, CD003125, 2003*
17. Vugt, M. V., Wilairatana, P., Gemperli, B., Gathmann, I., Phaipun, L., Brockman, A., Luxemburger, C., White, N. J., Nosten, F. and Looareesuwan, S. (1999) Efficacy of six doses of artemether-lumefantrine (benflumetol) in multidrug-resistant Plasmodium *falciparum* malaria. *Am. J. Trop. Med. Hyg.* **60**: 936-942.
18. van Vugt, M., Looareesuwan, S., Wilairatana, P., McGready, R., Villegas, L., Gathmann, I., Mull, R., Brockman, A., White, N. J. and Nosten, F.(2000) Artemether-lumefantrine for the treatment of multidrug-resistant falciparum malaria.*Trans. R. Soc. Trop. Med. Hyg.* **94**: 545-548.
19. Falade, C, Makanga, M, Premji, Z, Ortmann, CE, Stockmeyer, M, de Palacios, PI. (2005) Efficacy and safety of artemether-lumefantrine (Coartem) tablets (six-dose regimen) in African infants and children with acute, uncomplicated falciparum malaria. *Trans. R. Soc.Trop. Med. Hyg.* **99**: 459-67.
20. Rwagacondo CE, Karema C, Mugisha V, Erhart A, Dujardin JC, Van Overmeir C, Ringwald P, D’Alessandro U. (2004) Is amodiaquine failing in Rwanda? Efficacy of amodiaquine alone and combined with artesunate in children with uncomplicated malaria. *Trop. Med. Int. Health.* **9**:1091-1098.
21. Denis, M. B., Davis, T. M., Hewitt, S., Incardona, S., Nimol, K., Fandeur, T., Poravuth, Y., Lim, C. and Socheat, D. (2002) Efficacy and safety of dihydroartemisinin-piperaquine (Artekin) in Cambodian children and adults with uncomplicated falciparum malaria. *Clin. Infect. Dis.* **35**: 1469-1476.
22. Djimde A, Doumbo OK, Cortese JF, Kayentao K, Doumbo S, Diourte Y, Dicko A, Su XZ, Nomura T, Fidock DA, Wellems TE, Plowe CV, Coulibaly D: A molecular marker for chloroquine-resistant falciparum malaria. N Engl J Med 2001, 344:257-263.
23. Warhurst DC: Drug resistance in Plasmodium falciparum malaria. Infection 1999, 27(Suppl 2):S55-58.
24. Fidock DA, Nomura T, Talley AK, Cooper RA, Dzekunov SM, Ferdig MT, Ursos LM, Sidhu AB, Naude B, Deitsch KW, Su XZ, Wootton JC, Roepe PD, Wellems TE: Mutations in the P. falciparum digestive vacuole transmembrane protein PfCRT and evidence for their role in chloroquine resistance. Mol Cell 2000, 6:861-871.
25. Ouellette M: Biochemical and molecular mechanisms of drug resistance in parasites. Trop Med Int Health 2001, 6:874-882.
26. Wellems TE, Plowe CV: Chloroquine-resistant malaria. J Infect Dis 2001, 184:770-776.
27. Bzik DJ, Li WB, Horii T, Inselburg J: Molecular cloning and sequence analysis of the Plasmodium falciparum dihydrofolate reductase-thymidylate synthase gene. Proc Natl Acad Sci USA 1987, 84:8360-8364.
28. Brooks DR, Wang P, Read M, Watkins WM, Sims PF, Hyde JE: Sequence variation of the hydroxymethyldihydropterin pyrophosphokinase: dihydropteroate synthase gene in lines of the human malaria parasite, Plasmodium falciparum, with differing resistance to sulfadoxine. Eur J Biochem 1994, 224:397-405.
29. Kublin JG, Dzinjalamala FK, Kamwendo DD, Malkin EM, Cortese JF, Martino LM, Mukadam RA, Rogerson SJ, Lescano AG, Molyneux ME, Winstanley PA, Chimpeni P, Taylor TE, Plowe CV: Molecular markers for failure of sulfadoxine-pyrimethamine and chlorproguanil-dapsone treatment of Plasmodium falciparum malaria. J Infect Dis 2002, 185:380-388.
30. Happi CT, Gbotosho GO, Folarin OA, Bolaji OM, Sowunmi A, Kyle DE, Milhous W, Wirth DF, Oduola AM: Association between mutations in Plasmodium falciparum chloroquine resistance transporter and P. falciparum multidrug resistance 1 genes and in vivo amodiaquine resistance in P. falciparum malaria-infected children in Nigeria. Am J Trop Med Hyg 2006, 75:155-161.
31. Schwobel B, Alifrangis M, Salanti A, Jelinek T: Different mutation patterns of atovaquone resistance to Plasmodium falciparum in vitro and in vivo: rapid detection of codon 268 polymorphisms in the cytochrome b as potential in vivo resistance marker. Malar J 2003, 2:5.
32. Musset L, Bouchaud O, Matheron S, Massias L, Le Bras J: Clinical atovaquone-proguanil resistance of Plasmodium falciparum associated with cytochrome b codon 268 mutations. Microbes Infect 2006, in press.
33. Wichmann O, Muehlberger N, Jelinek T, Alifrangis M, Peyerl-Hoffmann G, Muhlen M, Grobusch MP, Gascon J, Matteelli A, Laferl H, Bisoffi Z, Ehrhardt S, Cuadros J, Hatz C, Gjorup I, McWhinney P, Beran J, da Cunha S, Schulze M, Kollaritsch H, Kern P, Fry G, Richter J, European Network on Surveillance of Imported Infectious Diseases: Screening for mutations related to atovaquone/proguanil resistance in treatment failures and other imported isolates of Plasmodium falciparum in Europe. J Infect Dis 2004, 190:1541-1546.
34. Sisowath C, Stromberg J, Martensson A, Msellem M, Obondo C, Bjorkman A, Gil JP: In vivo selection of Plasmodium falciparum pfmdr1 86N coding alleles by artemether-lumefantrine (Coartem). J Infect Dis 2005, 191(6):1014-1017.
35. Duraisingh MT, Jones P, Sambou I, von Seidlein L, Pinder M, Warhurst DC: The tyrosine-86 allele of the pfmdr1 gene of Plasmodium falciparum is associated with increased sensitivity to the anti-malarials mefloquine and artemisinin. Mol Biochem Parasitol 2000, 108:13-23.
36. Pickard, A. L., C. Wongsrichanalai, A. Purfield, D. Kamwendo, K. Emery, C. Zalewski, F. Kawamoto, R. S. Miller, and S. R. Meshnick. 2003 . Resistance to antimalarials in Southeast Asia and genetic polymorphisms in pfmdr1. Antimicrob. Agents Chemother. 47:2418-2423.
37. Price, R. N., A. C. Uhlemann, A. Brockman, R. McGready, E. Ashley, L. Phaipun, R. Patel, K. Laing, S. Looareesuwan, N. J. White, F. Nosten, and S. Krishna. 2004 . Mefloquine resistance in Plasmodium falciparum and increased pfmdr1 gene copy number. Lancet 364:438-447.
38. Reed, M. B., K. J. Saliba, S. R. Caruana, K. Kirk, and A. F. Cowman. 2000 . Pgh1 modulates sensitivity and resistance to multiple antimalarials in Plasmodium falciparum. Nature 403:906-909.
39. Sidhu, A. B., S. G. Valderramos, and D. A. Fidock. 2005 . pfmdr1 mutations contribute to quinine resistance and enhance mefloquine and artemisinin sensitivity in Plasmodium falciparum. Mol. Microbiol. 57:913-926.
40. Sisowath, C., J. Stromberg, A. Martensson, M. Msellem, C. Obondo, A. Bjorkman, and J. P. Gil. 2005 . In vivo selection of Plasmodium falciparum pfmdr1 86N coding alleles by artemether-lumefantrine (Coartem). J. Infect. Dis. 191:1014-1017.
41. Christian, D., Samuel, L. N., Bryan, G.,1 Philip J. R.., Grant, D. Selection of Plasmodium falciparum pfmdr1 Alleles following Therapy with Artemether-Lumefantrine in an Area of Uganda where Malaria Is Highly Endemic. Antimicrob Agents Chemother. 2006 May; 50(5): 1893–1895.
42. Hasifa B, Adoke Y, Moses RK, Ambrose T, Kristin B, Nathan B, John BR, Philip JR, Fred WM, Grant D, Sarah GS. “Artemisinin combination therapies for treatment of uncomplicated malaria in Uganda” PLOS Clinical Trials. 2006 May e7.

# APPENDICES

**APPENDIX I**

Guidelines for Grading Patient Symptoms, signs and laboratory findings.

Table A. Guidelines for Grading Patient Symptoms.

|  | **Grade 1**  **MILD** | **Grade 2**  **MODERATE** | **Grade 3**  **SEVERE** | **Grade 4**  **LIFE THREATENING** |
| --- | --- | --- | --- | --- |
| **Subjective fever in the past 24 h** | N/A | Present (Yes) | N/A | N/A |
| ***Weakness*** | Mild decrease in activity; For children – weak, but still playing | Moderate decrease in activity; For children – weak, and playing limited | Not participating in usual activities; For children – not playing | Prostration |
| **Muscle and/or joint aches*** | Mild and/or localized complaints | Diffuse complaints | Objective weakness; function limited | N/A |
| **Headache*** | Mild, no treatment required | Transient, moderate; treatment required | Severe, constant; requires narcotic therapy | Intractable; requires repeated narcotic therapy |
| **Anorexia** | Decreased appetite, but still taking solid food | Decreased appetite, avoiding solid food but taking liquids | Appetite very decreased; Refusing to breast feed, no solids or liquids taken (< 2 years < 12 hr; > 2 years < 24 hr) | Appetite very decreased; Refusing to breast feed, no solids or liquids taken (< 2 years > 12 hr; > 2 years > 24 hr) |
| **Nausea*** | Mild, transient feeling of impending vomiting; maintains reasonable intake | Moderate and/or constant feeling of impending vomiting; intake decreased | Severe, constant feeling of impending emesis; intake decreased significantly | N/A |
| **Vomiting** | 1 episode per day | 2-3 episodes per day | Orthostatic hypotension or IV fluids required | Hypotensive shock or physiological consequences requiring IV fluid therapy |
| **Abdominal pain*** | Mild (1-3 on a scale of 1 to 10) | Moderate (4-6 on a scale of 1 to 10) | Moderate to severe (> 7 on a scale of 1 to 10) | Severe – hospitalization for treatment |
| **Diarrhea** | Transient 3-4 loose stools/day | 5-7 loose stools/day | Orthostatic hypotension or > 7 loose stools/day or IV fluids required | Hypotensive shock or physiological consequences requiring IV fluid therapy |
| **Cough** | Transient / intermittent | Persistent / constant | Uncontrolled | Cyanosis, stridor, severe shortness of breath |
| **Pruritis** | Transient pruritis | Pruritis that disturbs sleep | Severe, constant pruritis, sleep disturbed | N/A |
| **Tinnitus*** | Mild, transient ringing or roaring sound | Moderate, persistent ringing or roaring sound | Severe ringing or roaring sound with associated hearing loss | N/A |
| **Behavioural changes** | Mild difficulty concentrating; mild confusion or agitation; activities of daily living unaffected; no treatment | Moderate confusion or agitation; some limitation of activities of daily living; minimal treatment | Severe confusion or agitation; Needs assistance for activities of daily living; therapy required | Toxic psychosis; hospitalization for treatment |
| **“Flu”**  **(viral URI)** | Mild nasal congestion, mild rhinorrhea | Moderate nasal congestion, moderate rhinorrhea | N/A | N/A |
| **Allergic reaction** | N/A | N/A | Urticaria | Severe urticaria  anaphylaxis, angioedema |
| **Convulsion** | N/A | N/A | Localized or generalized seizure | Status epilepticus |
| *** Assess only in children > 3 years of age. Answer N/A for younger children and those unable to answer.** | | | | |

Reference – Based on WHO Toxicity Grading Scale for Determining the Severity of Adverse Events

**Table B. Guidelines for Physical Examination**

| **Dehydration** | Assess skin touch and turgor, mucous membranes, eyes, crying, fontanelle, pulse, urine output |
| --- | --- |
| **Jaundice** | Assess for yellowing of the sclera. Also evaluate the palpebral conjunctiva, lips, and skin. |
| **Chest** | Observe the rate, rhythm, depth, and effort of breathing. Check the patient’s colour for cyanosis.  The maximum acceptable respiratory rate by age: < 2 months = 60, 2-12 months = 50, 1-5 years = 40, above 5 years = 30.  Inspect the neck for the position of the trachea, for supraclavicular retractions, and for contraction of the sternomastoid or other accessory muscles during inspiration.  Auscultate the anterior and posterior chest for normal breath sounds and any adventitious sounds (crackles or rales, wheezes, and rhonchi). *Crackles are intermittent, non-musical, fine or coarse sounds that may be due to abnormalities of the lungs (pneumonia, fibrosis, early congestive heart failure) or airways (bronchitis or bronchiectasis). Wheezes are high-pitched and result from narrowed airways. Rhonchi are relatively low-pitched and suggest secretions in large airways.*  If abnormalities are identified, evaluate for transmitted voice sounds. In addition, palpate the chest to assess for tactile fremitus, and percuss the chest to assess for areas of dullness*. Normal, air-filled lungs emit predominantly vesicular breath sounds, transmit voice sounds poorly with “ee” = “ee”, and have no tactile fremitus. Airless lung, as in lobar pneumonia, emits bronchial breath sounds, transmits spoken words clearly with “ee” = “aay” (egophany), and has an increase in tactile fremitus.* |
| **Abdomen** | Inspection and auscultation of the abdomen. Listen for bowel sounds in the abdomen before palpating it. Palpate the abdomen in all 4 quadrants lightly and then deeply. Assess the size of the liver and spleen. To assess for peritoneal inflammation, look for localised and rebound tenderness, and voluntary or involuntary rigidity. |
| **Skin** | Inspect the skin for colour, turgor, moisture, and lesions. If lesions are present, note their location and distribution (diffuse or localised), arrangement (linear, clustered, annular, dermatomal), type (macules, papules, vesicles) and colour. |
| **Tablet test** | For children > 9 months of age, ask the patient to pick a tablet (or equivalent object) up off a flat surface using the thumb and index finger of their dominant hand*. This tests for co-ordination of the upper extremity assessing the function of the motor system, cerebellar system, vestibular system (for coordinating eye and body movements) and the sensory system, for position sense. When testing small children, be aware that they will likely attempt to put the object into their mouth.* |

**Table C. Grading Physical Examination Findings**

|  | **Grade 1**  **MILD** | **Grade 2**  **MODERATE** | **Grade 3**  **SEVERE** | **Grade 4**  **LIFE-THREATENING** |
| --- | --- | --- | --- | --- |
| **Temperature* (axillary)** | 37.5-37.9°C | 38.0-39.5°C | > 39.5°C | Sustained fever, equal or greater than 40.0°C for longer than 5 days |
| **Dehydration** | Less than 2 of the following:  Restless, irritable  Sunken eyes  Drinks eagerly, thirsty  Skin pinch goes back slowly | 2 of the following:  Restless, irritable  Sunken eyes  Drinks eagerly, thirsty  Skin pinch goes back slowly | Two of the following:  Lethargic or unconscious  Sunken eyes  Not able to drink or drinking poorly  Skin pinch goes back very poorly | Two of the following + shock:  Lethargic or unconscious  Sunken eyes  Not able to drink or drinking poorly  Skin pinch goes back very poorly |
| **Jaundice** | Slight yellowing of sclera and conjunctiva | Moderate yellowing of sclera and conjunctiva, yellowing of mucous membranes | Severe yellowing of sclera and conjunctiva, yellowing of skin | N/A |
| **Chest** | Mildly increased RR (for age, temperature), transient or localised adventitious sounds | Moderately increased RR, diffuse or persistent adventitious sounds | Rapid RR (< 2 months > 60, 2-12 months > 50, 1-5 years > 40, adults > 30)* nasal flaring, retractions | Cyanosis |
| **Abdomen** | Normal bowel sounds, mild localised tenderness, and/or liver palpable 2-4 cm below the right costal margin (RCM), and/or spleen palpable, and/or umbilical hernia present | Normal or mildly abnormal bowel sounds, moderate or diffuse tenderness; and/or mild to moderately enlarged liver (4-6 cm below the RCM) and/or spleen palpable up to half-way between umbilicus and symphysis pubis | Severely abnormal bowel sounds, severe tenderness to palpation. Evidence of peritoneal irritation and/or significant enlargement of liver (> 6 cm below the RCM) and/or spleen palpable beyond half-way between umbilicus and symphysis pubis | Absent bowel sounds. Involuntary rigidity |
| **Skin†** | Localised rash, erythema, or pruritis | Diffuse, maculopapular rash, dry desquamation | Vesiculation, moist desquamation, or ulceration | Exfoliative dermatitis, mucous membrane involvement or erythema multiforme or suspected Stevens-Johnson or necrosis requiring surgery |

|  | **Grade 1**  **MILD** | **Grade 2**  **MODERATE** | **Grade 3**  **SEVERE** | **Grade 4**  **LIFE-THREATENING** |
| --- | --- | --- | --- | --- |
| **Hearing** | *< 4 years: N/A*  > 4 years: Decreased hearing in one ear | *< 4 years: N/A*  > 4 years: Decreased hearing in both ears or severe impairment in one ear | *< 4 years: Any evidence of hearing impairment*  > 4 years: Severe impairment in both ears | N/A |
| **Tablet test** | Difficulty grasping tablet but able to pick up | Unable to pick up tablet without dropping | Unable to grasp tablet | N/A |
| **Clinical symptoms / sign *(not otherwise specified)*** | No treatment required; monitor condition | Treatment required | Requires treatment and possible hospitalisation | Requires active medical intervention, hospitalisation, or hospice care |

Reference – The Harriet Lane Handbook, 15th edition, 2000

† Reference – WHO Toxicity Grading Scale for Determining the Severity of Adverse Events

**TABLE D. Guidelines for Grading of Laboratory results**

|  | **Grade 1**  **MILD** | **Grade 2**  **MODERATE** | **Grade 3**  **SEVERE** | **Grade 4**  **LIFE-THREATENING** |
| --- | --- | --- | --- | --- |
| **Absolute neutrophil count* *(/****mm^3^****)*** | 750-1200 | 400-749 | 250-399 | < 250 |
| **Hemoglobin *(****g/dL)* | 9.0 – 9.9 | 7.0 – 8.9 | 5.0 – 6.9 | < 5.0 |
| **Platelets *(/****mm^3^****)**** | N/A | 50,000-75,000 | 25,000-49,999 | < 25,000 |
| **ALT *(U/L)***** | 1.1-4.9 x ULN  (50 – 224) | 5.0-9.9 x ULN  (225 – 449) | 10.0-15.0 x ULN  (450 – 675) | > 15.0 x ULN  (> 675) |
| **Bilirubin (*mg/dL)**** | 1.1-1.9 x ULN  (1.3 - 2.3) | 2.0-2.9 x ULN  (2.4 - 3.5) | 3.0-7.5 x ULN  (3.6 - 9.0) | > 7.5 x ULN  (> 9.0) |
| **Creatinine *(mg/dl))****  **Age < 2 years** | 0.6-0.8 | 0.9-1.1 | 1.2-1.5 | > 1.5 |
| **Creatinine *(mg/dl))****  **Age > 2 years** | 0.7-1.0 | 1.1-1.6 | 1.7-2.0 | > 2.0 |
| **Laboratory values** *(not otherwise specified)* | Abnormal but requiring no immediate intervention; follow | Sufficiently abnormal to require evaluation as to causality and perhaps mild therapeutic intervention | Sufficiently severe to require evaluation and treatment | Life-threatening severity; requires immediate evaluation, treatment, and usually hospitalization |

*Reference – DMID Pediatric Toxicity Tables, May 2001

** Reference – DAIDS Pediatric guidelines December 2004

**APPENDIX II***

**Table A. Dihydroartemisinine-piperaquine (DHAPQ)**

DHAPQ tablets are green film coated intended for oral use and contain 20/160mg or 40/320mg of dihydroartemisinin (DHA) and piperaquine phosphate (PQ) respectively.

DHAPQ 20/160 mg Tablets Components

Composition

One tablet contains

| Component | Amount (mg) |
| --- | --- |
| Piperaquine Phosphate (ST 3073) | 160 |
| Dehydroartemisinine (ST 3074) | 20 |
| Starch Maize | 34.5 |
| Dextrin | 26.4 |
| Hydroxypropylmethylcellulose | 2.4 |
| Sodium carboxymethylcellulose | 11.8 |
| Magniesum Stereate | 2.4 |
| OY-31074 Opadry Green coating | 7.5 |

The composition of OY-31074 Opadry coating preparation is

| Component | (%w/w) |
| --- | --- |
| Hydroxypropylmethylcellulose | 62.501 |
| Titanium Dioxide | 25.450 |
| Macrogol 400 | 6.249 |
| FD&Blue #1 Brilliant blue A1 lake | 0.400 |
| FD&Blue #2 Indigo Carmine A1 lake | 0.400 |
| FD&C Yellow #5 Tartrazine A1 lake | 5.000 |

DHAPQ 40/320 mg Tablets Components

Composition

One tablet contains

| Component | Amount (mg) |
| --- | --- |
| Piperaquine Phosphate (ST 3073) | 320 |
| Dehydroartemisinine (ST 3074) | 40 |
| Starch Maize | 69 |
| Dextrin | 52.8 |
| Hydroxypropylmethylcellulose | 4.8 |
| Sodium carboxymethylcellulose | 23.6 |
| Magniesum Stereate | 4.8 |
| OY-31074 Opadry Green coating | 15 |

The composition of OY-31074 Opadry coating preparation is the same as above.

Dihydroartemisinin will be given daily. The number of tablets per day is reported in brackets. One tablet of DHAPQ (pediatric tablets) contains 20/40 mg of DHA and 160/320 mg of PPQ.

| **Weight in kg** | **mg of DHA to be given daily** | **Dose of DHA as mg/kg/d** |
| --- | --- | --- |
| 5 - 6 | 10 (1/2 tablet with 20 mg) | 1.67 - 2.5 |
| 7 - 12 | 20 (1 tablet with 20 mg) | 1.67 - 2.86 |
| 13 - 23 | 40 (1 tablet with 40 mg) | 1.74 - 3.08 |
| 24 - 35 | 80 (2 tablets with 40 mg) | 2.29 - 3.33 |

*One tablet of DHAPQ contains 40 mg of DHA and 320 mg of PPQ for adult patients.*

Piperaquine will be given daily. The number of tablets per day is reported in brackets. One tablet of DHAPQ (pediatric tablets) contains 20/40 mg of DHA and 160/320 mg of PPQ.

| **Weight in kg** | **mg of PPQ to be given daily** | **Dose of PPQ as mg/kg/d** |
| --- | --- | --- |
| 5 - 6 | 80 (1/2 tablet with 160 mg) | 13.36 - 20 |
| 7 - 12 | 160 (1 tablet with 160 mg) | 13.36 – 22.88 |
| 13 - 23 | 320 (1 tablet with 320 mg) | 13.92 – 24.64 |
| 24 - 35 | 640 (2 tablets with 320 mg) | 18.32 – 26.4 |

*One tablet of DHAPQ contains 40 mg of DHA and 320 mg of PPQ for adult patients.*

**Table B:** Coartem® dose based on body weight will be given daily. Tablets containing 20 mg of Artemether and 120 mg of Lumefantrine.

| **Weight in kg** | **Number of tablet per dose** |
| --- | --- |
| 5 to < 15 kg | 1 tablet per dose |
| 15 to < 25 kg | 2 tablets per dose |
| 25 to < 35 kg | 3 tablets per dose |

**Table C:** Quinine dose based on body weight will be given daily. Quinine sulphate will be provided as tablets of 300 mg (International Dispensary Association [IDA], Amsterdam, The Netherlands)

| **Weight in kg** | **Number of tablet per dose** | **Dose of quinine as mg/kg/day** |
| --- | --- | --- |
| 5 to < 7 kg | ¼ tablet per dose | 15 - 10 mg/kg |
| 12 to < 15 kg | ½ tablets per dose | 13 – 10 mg/kg |
| 16 to < 22 kg | ¾ tablets per dose | 14 – 10 mg/kg |
| 23 to < 30 kg | 1 tablets per dose | 13 – 10 mg/kg |
| 31 to < 35 kg | 1¼ tablets per dose | 12 – 10 mg/kg |

**APPENDIX III**

**WORLD MEDICAL ASSOCIATION DECLARATION OF HELSINKI**

**Ethical Principles for Medical Research Involving Human Subjects**

**Recommendations guiding medical physicians**

**in biomedical research involving human subjects**

Adopted by the 18th WMA General Assembly

Helsinki, Finland, June 1964

and amended by the

29th WMA General Assembly, Tokyo, Japan, October 1975

35th WMA General Assembly, Venice, Italy, October 1983

41st WMA General Assembly, Hong Kong, September 1989

48th WMA General Assembly, Somerset West, Republic of South Africa, October 1996

and the

52nd WMA General Assembly, Edinburgh, Scotland, October 2000

Note of Clarification on Paragraph 29 added by the WMA General Assembly, Washington 2002.

**A. INTRODUCTION**

1. The World Medical Association has developed the Declaration of Helsinki as a statement of ethical principles to provide guidance to physicians and other participants in medical research involving human subjects. Medical research involving human subjects includes research on identifiable human material or identifiable data.

2. It is the duty of the physician to promote and safeguard the health of the people. The physician's knowledge and conscience are dedicated to the fulfillment of this duty.

3. The Declaration of Geneva of the World Medical Association binds the physician with the words, "The health of my subject will be my first consideration," and the International Code of Medical Ethics declares that, "A physician shall act only in the subject's interest when providing medical care which might have the effect of weakening the physical and mental condition of the subject."

4. Medical progress is based on research which ultimately must rest in part on experimentation involving human subjects.

5. In medical research on human subjects, considerations related to the well-being of the human subject should take precedence over the interests of science and society.

6. The primary purpose of medical research involving human subjects is to improve prophylactic, diagnostic and therapeutic procedures and the understanding of the etiology and pathogenesis of disease. Even the best proven prophylactic, diagnostic, and therapeutic methods must continuously be challenged through research for their effectiveness, efficiency, accessibility and quality.

7. In current medical practice and in medical research, most prophylactic, diagnostic and therapeutic procedures involve risks and burdens.

8. Medical research is subject to ethical standards that promote respect for all human beings and protect their health and rights. Some research populations are vulnerable and need special protection. The particular needs of the economically and medically disadvantaged must be recognized. Special attention is also required for those who cannot give or refuse consent for themselves, for those who may be subject to giving consent under duress, for those who will not benefit personally from the research and for those for whom the research is combined with care.

9. Research Investigators should be aware of the ethical, legal and regulatory requirements for research on human subjects in their own countries as well as applicable international requirements. No national ethical, legal or regulatory requirement should be allowed to reduce or eliminate any of the protections for human subjects set forth in this Declaration.

**B. BASIC PRINCIPLES FOR ALL MEDICAL RESEARCH**

10. It is the duty of the physician in medical research to protect the life, health, privacy, and dignity of the human subject.

11. Medical research involving human subjects must conform to generally accepted scientific principles, be based on a thorough knowledge of the scientific literature, other relevant sources of information, and on adequate laboratory and, where appropriate, animal experimentation.

12. Appropriate caution must be exercised in the conduct of research which may affect the environment, and the welfare of animals used for research must be respected.

13. The design and performance of each experimental procedure involving human subjects should be clearly formulated in an experimental protocol. This protocol should be submitted for consideration, comment, guidance, and where appropriate, approval to a specially appointed ethical review committee, which must be independent of the Investigator, the Sponsor or any other kind of undue influence. This independent committee should be in conformity with the laws and regulations of the country in which the research experiment is performed. The committee has the right to monitor ongoing trials. The researcher has the obligation to provide monitoring information to the committee, especially any serious adverse events. The researcher should also submit to the committee, for review, information regarding funding, Sponsors, institutional affiliations, other potential conflicts of interest and incentives for subjects.

14. The research protocol should always contain a statement of the ethical considerations involved and should indicate that there is compliance with the principles enunciated in this Declaration.

15. Medical research involving human subjects should be conducted only by scientifically qualified persons and under the supervision of a clinically competent medical person. The responsibility for the human subject must always rest with a medically qualified person and never rest on the subject of the research, even though the subject has given consent.

16. Every medical research project involving human subjects should be preceded by careful assessment of predictable risks and burdens in comparison with foreseeable benefits to the subject or to others. This does not preclude the participation of healthy volunteers in medical research. The design of all studies should be publicly available.

17. Physicians should abstain from engaging in research projects involving human subjects unless they are confident that the risks involved have been adequately assessed and can be satisfactorily managed. Physicians should cease any investigation if the risks are found to outweigh the potential benefits or if there is conclusive proof of positive and beneficial results.

18. Medical research involving human subjects should only be conducted if the importance of the objective outweighs the inherent risks and burdens to the subject. This is especially important when the human subjects are healthy volunteers.

19. Medical research is only justified if there is a reasonable likelihood that the populations in which the research is carried out stand to benefit from the results of the research.

20. The subjects must be volunteers and informed participants in the research project.

21. The right of research subjects to safeguard their integrity must always be respected. Every precaution should be taken to respect the privacy of the subject, the confidentiality of the subject's information and to minimize the impact of the study on the subject's physical and mental integrity and on the personality of the subject.

22. In any research on human beings, each potential subject must be adequately informed of the aims, methods, sources of funding, any possible conflicts of interest, institutional affiliations of the researcher, the anticipated benefits and potential risks of the study and the discomfort it may entail. The subject should be informed of the right to abstain from participation in the study or to withdraw consent to participate at any time without reprisal. After ensuring that the subject has understood the information, the physician should then obtain the subject's freely-given informed consent, preferably in writing. If the consent cannot be obtained in writing, the non-written consent must be formally documented and witnessed.

23. When obtaining informed consent for the research project the physician should be particularly cautious if the subject is in a dependent relationship with the physician or may consent under duress. In that case the informed consent should be obtained by a well-informed physician who is not engaged in the investigation and who is completely independent of this relationship.

24. For a research subject who is legally incompetent, physically or mentally incapable of giving consent or is a legally incompetent minor, the Investigator must obtain informed consent from the legally authorized representative in accordance with applicable law. These groups should not be included in research unless the research is necessary to promote the health of the population represented and this research cannot instead be performed on legally competent persons.

25. When a subject deemed legally incompetent, such as a minor child, is able to give assent to decisions about participation in research, the Investigator must obtain that assent in addition to the consent of the legally authorized representative.

26. Research on individuals from whom it is not possible to obtain consent, including proxy or advance consent, should be done only if the physical/mental condition that prevents obtaining informed consent is a necessary characteristic of the research population. The specific reasons for involving research subjects with a condition that renders them unable to give informed consent should be stated in the experimental protocol for consideration and approval of the review committee. The protocol should state that consent to remain in the research should be obtained as soon as possible from the individual or a legally authorized surrogate.

27. Both authors and publishers have ethical obligations. In publication of the results of research, the Investigators are obliged to preserve the accuracy of the results. Negative as well as positive results should be published or otherwise publicly available. Sources of funding, institutional affiliations and any possible conflicts of interest should be declared in the publication. Reports of experimentation not in accordance with the principles laid down in this Declaration should not be accepted for publication.

**C. ADDITIONAL PRINCIPLES FOR MEDICAL RESEARCH COMBINED WITH MEDICAL CARE**

28. The physician may combine medical research with medical care, only to the extent that the research is justified by its potential prophylactic, diagnostic or therapeutic value. When medical research is combined with medical care, additional standards apply to protect the subjects who are research subjects.

29. The benefits, risks, burdens and effectiveness of a new method should be tested against those of the best current prophylactic, diagnostic, and therapeutic methods. This does not exclude the use of placebo, or no treatment, in studies where no proven prophylactic, diagnostic or therapeutic method exists.

30. At the conclusion of the study, every subject entered into the study should be assured of access to the best proven prophylactic, diagnostic and therapeutic methods identified by the study.

31. The physician should fully inform the subject which aspects of the care are related to the research. The refusal of a subject to participate in a study must never interfere with the subject-physician relationship.

32. In the treatment of a subject, where proven prophylactic, diagnostic and therapeutic methods do not exist or have been ineffective, the physician, with informed consent from the subject, must be free to use unproven or new prophylactic, diagnostic and therapeutic measures, if in the physician's judgment it offers hope of saving life, re-establishing health or alleviating suffering. Where possible, these measures should be made the object of research, designed to evaluate their safety and efficacy. In all cases, new information should be recorded and, where appropriate, published. The other relevant guidelines of this Declaration should be followed.

**＊FOOTNOTE:** **Note of Clarification on Paragraph 29 of the WMA Declaration of Helsinki**

The WMA hereby reaffirms its position that extreme care must be taken in making use of a placebo-controlled trial and that in general this methodology should only be used in the absence of existing proven therapy. However, a placebo-controlled trial may be ethically acceptable, even if proven therapy is available, under the following circumstances:

Where for compelling and scientifically sound methodological reasons its use is necessary to determine the efficacy or safety of a prophylactic, diagnostic or therapeutic method; or

Where a prophylactic, diagnostic or therapeutic method is being investigated for a minor condition and the patients who receive placebo will not be subject to any additional risk of serious or irreversible harm.

**APPENDIX IV**

**RESEARCH PARTICIPANT INFORMED CONSENT FORM**

STUDY TITLE

**Evaluation of the best approach to retreating recurrent malaria in Ugandan children.**

Please read the background information and informed consent form carefully. The background information explains your rights and our responsibilities to you. If you have any questions concerning the study please do not hesitate to ask any of the doctors. Before you decide, it is important for you to understand why the research is being done and what it will involve. You will be given a copy of this signed document (Informed Consent Form) to take home with you.

**YOU MUST KEEP THIS BACKGROUND INFORMATION WITH YOU THROUGHOUT THE STUDY PERIOD.**

**PURPOSE OF THE STUDY**

This research study is being done to learn more about the treatment of malaria. This is a nested study and you have already consented to participate in the main study. We are carrying out the research study to compare different medicines for the treatment of mild recurrent malaria. The medicines we are studying are: dihydroartemisinin-piperaquine, arthemeter-lumefantrine, and quinine. All these drugs are active against malaria. With this study we want to find out their relative value in curing malaria in terms of efficacy and safety. Two hundred and sixty patients from this health centre will participate in this study.

**HOW THE STUDY IS DONE**

The child under your care will be treated for malaria with one of the above study medicines. After the treatment, your child will be actively followed for 28 days to see if the malaria infection is completely cured. If your child is not completely cured by the study medicines, s/he will then be given treatment according to the standard practice in the country. The study medicine that your child will receive will be determined by a process of randomization. Randomization means that your child will receive one of the medicines studied by chance. You are being asked to allow your child (child under your care in the case of a legal guardian), to participate in this study. Your child will be actively followed up for 28 days or until such time as you or the study doctors decide that your child should no longer participate in the study. You can choose to withdraw your consent to participate in the study any time and without influencing the medical attention your child may need. The study may be discontinued by the sponsor at any time, and for any reason.

**PROCEDURES**

- 1. The study doctors will examine your child today.
  2. A blood sample will be collected. A small amount of blood will be taken by fingerprick to examine for malaria parasites, to measure the blood count, to store blood samples on filter paper for future laboratory tests that will not impact on the health care of your child.
  3. If the diagnosis of malaria is confirmed, and your child is eligible for the study, treatment with either dihydroartemisinin + piperaquine (DHA+PPQ), arthemeter + lumefantrine (A+L), or quinine will be given at the clinic. Children randomised to dihydroartemisinin + piperaquine (DHA+PPQ) or arthemeter + lumefantrine (A+L) will receive treatment during the first 3 days. Those randomised to quinine will receive treatment during the first 7 days. Treatment administration during the first 3 days will be directly observed. This means that your child may be hospitalised during the first 3 days of the study. For children on quinine the remaining two doses for the day shall be administered in the clinic under direct supervision and the third dose for the day shall be administered by the parents at home.
  4. You will be asked to return to the clinic at least 7 more times over the next month so that the success of the treatment can be judged. At each of the follow-up visits, your child will be examined by the study doctors and, a small amount of blood will taken by fingerprick to examine for malaria parasites, measure haemoglobin and to save on filter paper.
  5. If in any you miss an appointment, the home health visitor will visit your child at your home to find out why you missed the appointment and bring your child to the clinic for assessment.
  6. If, at any time, during the 28 days of follow up the child develops malaria again, those treated with dihydroartemisinin + piperaquine (DHA+PPQ) or arthemeter + lumefantrine (A+L) will be given quinine while those treated with quinine will receive artemether + clindamycin.
  7. There will be someone at the study clinic every day from 8:00 am to 5:00 pm and at night. You can come to the clinic for evaluation anytime that your child is ill during the next 28 days.

RISKS AND DISCOMFORTS

1. Side effects following treatment with the study medications could occur. Generally, side effects (nausea, headache, dizziness…) are expected to be mild and short-lived.

Your child will be monitored closely after receiving treatment for malaria with the study medications for any possible side effects of the drugs and will receive appropriate medical care for any problem that happens during the course of the study.

1. Randomization: Your child will be assigned to a treatment group by chance. The treatment your child receives may prove to be less effective or to have more side effects than the other study treatments or than other available treatments. This will not be known until after the study is completed.
2. Severe malaria: Your child may develop malaria that is severe even after receiving treatment with study medications. If your child shows any evidence of severe malaria (including persistent vomiting, low blood (anaemia), convulsions, confusion, or coma) treatment with the usual standard of care will be given and your child will be referred for possible admission to hospital.
3. Blood draws: The risks of drawing blood from a fingerprick include temporary discomfort from the needle stick, bruising, skin infection, and fainting. The amount of blood removed will be too small to affect your child’s health.
4. Unknown Risks: The research treatments may have side effects that no one knows about yet. The researchers will let you know if they learn anything that might make you change your mind about your child’s participation in the study.
5. Confidentiality: Participation in research may involve a loss of privacy, but information about your child will be handled as confidentially as possible. Medical information related to malaria will be collected on your child, but only the people working on the study will see it. Anyone assigned to review this study will be granted direct access to your child's medical records, if necessary, for verification of the study procedures and data. Records will be kept as confidential as possible.

BENEFITS

1. The potential benefit to your child is that the treatment received may prove to be more effective than the other study treatments or than other available treatments, although this cannot be guaranteed.
2. Your child will receive clinical care from the medical officers and nurses of the project staff in the study clinic. This will include care for unscheduled sick visits.
3. The knowledge gained from this study will help your country in determining the best treatment for recurrent uncomplicated malaria.

COST/PAYMENT

After enrolment in the study, you will not be charged for clinic visits or treatment. Your child will not be paid for participation in the study. We will reimburse any transport costs incurred for clinic visits and meals will be provided when your child is admitted for observation and treatment administration.

**ALTERNATIVES TO PARTICIPATION**

Your child’s participation in this study is completely voluntary. If you decide that you do not want to participate in the study or decide to withdraw your child from the study at any time and for any reason, this will not affect your child’s care at the outpatient department, where standard care for all medical problems is available. During the study, you will be informed promptly of any new information that may influence your willingness to continue participation in the study.

**CONSEQUENCES OF WITHDRAWAL**

Should you decide to withdraw your child from the study before your child has finished the course of study medicines, then your child will receive the local standard treatment for malaria from the study team, but after the standard treatment has been given, medical care will no longer be provided by the study team. If the child is withdrawn from the study after completion of the course of study medicines, then no further care will be provided by the study team.

**USE OF THE RESULTS**

The findings from this study may be published in a medical journal. The study participants will not be identified by name. After the study is completed, you may request an explanation of the study results.

**TREATMENT AND COMPENSATION FOR INJURY**

If you are injured or have questions about injuries as a result of being in the study, please contact the doctors in the study clinic. The services at the public health facility will be open to you in case of any such injury.

**VOLUNTARY PARTICIPATION**

Participation in this study is entirely voluntary. You have the right to refuse your child’s participation or to withdraw at any point in this study without negative consequences or loss of benefits to which you and your child are otherwise entitled.

**IMPLICATION OF YOUR SIGNATURE OR THUMBPRINT**

If you give consent for your child to participate in this study, you should sign or place your thumbprint in the consent form. Your signature or thumbprint below means that you understand the information given to you about your child’s participation in the study and in the consent form. You will also be asked to sign another copy of this informed consent form for documentation.

**CONSENT FORM**

**CONSENT FORM FOR PARTICIPATION IN RESEARCH PROJECTS**

**AND CLINICAL TRIALS**

**Study Title**

**Evaluation of the best approach to retreating recurrent malaria in Ugandan children.**

Principal Investigator: Professor Umberto D'Alessandro .

Address: Prince Leopold Institute of Tropical Medicine, Nationalestraat 155, B- 2000, Antwerp, Belgium

Contact number: 0032 3 247 6354

I, …………………………………. mother/father/legal representative declare that I have understood the objectives and purposes of this study. I agree that my child……………………………………………………………. participates in this study.

I am aware that I can withdraw my child from the study at any time without any consequence to my child or to me.

Name of parent/legal representative

Signature or Thumbprint * of parent/ legal representative Date/Time

*If the parent or guardian is unable to read and/or write, an impartial witness should be present during the informed consent discussion. After the written informed consent form is read and explained to the parent or guardian, and after they have orally consented to their child’s participation in the trial, and have either signed the consent form or provided their fingerprint, the witness should sign and personally date the consent form. By signing the consent form, the witness attests that the information in the consent form and any other written information was accurately explained to, and apparently understood by, the parent or guardian, and that informed consent was freely given by the parent or guardian.

Name of Person Witnessing Consent (printed)

Signature of Person Witnessing Consent Date/Time

**APPENDIX V**

**Criteria for Severe Malaria/Danger Signs**

**Severe Malaria**

- Unarousable coma *(if after convulsion, > 30 min)*
- Repeated convulsions *(> 2 within 24 h)*
  - Severe anaemia *(Hb < 5.0 g/dL)*
  - Respiratory distress *(laboured breathing at rest)*

**Danger Signs**

- Recent convulsions *(>1 within 24 h)*
- Altered consciousness  *(confusion)*
- Lethargy
  - Unable to drink or breast feed
  - Vomiting everything
  - Unable to stand/sit due to weakness

**APPENDIX VI. PARTICIPANT SELECTION AND ENROLLMENT**

Children recruited in the main study attending Health Facility (HF) with fever or history of fever

Send to laboratory

Screening thick blood smear

Negative smear Parasites present

Refer patient to clinician

Patient continues active follow up in main study

Complete Screening Form

No consent

Patient continues active follow up in main study

Complete Informed Consent Form

Verify inclusion/exclusion criteria

- Hypersensitivity to the study drugs.
- Severe malaria or danger signs
- Intercurrent illness

Complete Case Report Forms

Refer to the Laboratory

Refer to the Investigator/Study Nurse

Admit at the clinic or manage as outpatients for 3 or 7 days

Continue with scheduled follow-up.

**APPENDIX VII. CRITICAL STEPS.**

**Days 0 and 1**

Evaluate patient and complete Case Report form. Administer study medications.

**Day 2.**

Evaluate patient and complete Case Report Form. Administer study medications.

Collect thick blood smear and filter paper sample

**Day 3.** Evaluate patient and complete Case Report Form. Collect thick blood smear and filter paper sample.

Continue quinine treatment D3-D6.

**Day 7, 14, 21**. Evaluate patient and complete Case Report Form. Collect thick blood smear and filter paper sample

**Any Unscheduled Day (Day 4-27).**

Evaluate patient and complete Case Record Form. Collect thick blood smear with filter paper sample.

D2 Parasitaemia > D 0 parasitaemia

**ETF**

Treat with oral quinine or artemether (AM)+ clindamycin (CL).

**Severe disease or danger signs**

Do urgent thick smear, FP sample and Haemoglobin

If patient has

1. Temperature > 37.5^0^C with parasitaemia or

2. Parasite count >25% Day 0 count.

**ETF.** Treat with oral quinine or AM + CL

**Days 28**

Evaluate patient and complete Case Report Form. Collect thick blood smear and filter paper sample.

**Severe disease or danger signs**

Do urgent thick smear, FP sample and Haemoglobin

Negative smear

**Continue study**

**at your discretion.**

Negative smear

**Continue study at your discretion.**

Positive blood smear

**ETF**

**Give/ refer for IV quinine or artemether.**

Positive blood smear

**LCF**

**Give/ refer for IV Quinine or artemether.**

**ACPR** No parasitaemia on days 28 irrespective of fever history or temperature.

If patient has:

Temperature > 37.5^0^C with parasitaemia

**LCF**

Parasitaemia without fever

**LPF**

Treat with oral quinine or AM + CL

# APPENDIX IX: PATIENT SCREENING FORM

**STUDY SITE CODE:________ STUDY NUMBER: ___________________________**

UMSP SCREENING FORM

| **1. Names:** | **2. Date: *(dd/mm/yy)*** |
| --- | --- |
| **3. Age: _______years __________months.** | **4. Gender: M _______ F_______** |
| **5.Weight (kg)** |  |

*Weight for height should be at least 70% of the median NCHS/WHO reference.*

| **SCREENING selection criteria**  ***Patients who are 1 year-6 years of age and have a positive screening thick blood smear.*** | | |
| --- | --- | --- |
|  | **yes** | **NO** |
| 6. Not previously enrolled in this study |  |  |
| 7. Ability to participate in 28 day follow-up. |  |  |
| **EXCLUSION CRITERIA** | **NO** | **YES** |
| 8. History of serious side effects to study drugs  *If present, indicate drug / side effect:*   Artemether-lumefantrine:________________________   Dihydroartemisinin-piperaquine:______________________   Quinine:_____________________ |  |  |
| 9. Evidence of severe malaria / danger signs  *If “ YES” indicate criteria. If “NO”, leave blank.*   Unarousable coma *(if after convulsion, > 30 min)*   Recent convulsions *(> 1 within 24 h)*   Altered consciousness*(confusion, delirium,, coma)*   Lethargy   Unable to drink or breast feed   Vomiting (> twice in 24 hours)   Unable to stand/sit due to weakness   Severe anemia *(Hb < 5.0 g/dL)*   Respiratory distress *(labored breathing at rest)*   Jaundice *(yellow coloring of eyes)* |  |  |
| 10. Evidence of concomitant febrile illness  *If “YES”, indicate illness. If “NO”, leave blank.*   Pneumonia/RTI  Measles   Otitis Media  UTI   Gastroenteritis  Other:_________________ |  |  |
|  | **YES** | **NO** |
| 11. Provision of informed consent. |  |  |
| 12. Absence of persistent vomiting of study medications on day 0 |  |  |
| 13. *P.Falciparum mono infection* |  |  |

***If any of the responses fall into the shaded area, exclude the patient from the study***

# Appendix X. Adverse Event - Follow-up Report

**ADVERSE EVENT FORM – FOLLOW-UP REPORT**

| **1. Study**  **Number: U**|___|___|___|___|___| | **2. Day 0 Date:** |___|___|/|___|___|/|___|___|  ***day month year*** | **3. Treatment**  **Number: ______________** |
| --- | --- | --- |

| Date of follow-up: |___|___|/|___|___|/|___|___|  *day month year* | | | Study Day: | Temp: | |
| --- | --- | --- | --- | --- | --- |
| Progress Note:  _______________________________________________________  _______________________________________________________  _______________________________________________________  _______________________________________________________  _______________________________________________________  _______________________________________________________  _______________________________________________________  _______________________________________________________  _______________________________________________________  _______________________________________________________  _______________________________________________________  _______________________________________________________  _______________________________________________________ | | | Laboratory results / Other comments: | | |
| Date of follow-up: |___|___|/|___|___|/|___|___|  *day month year* | | | Study Day: | | Temp: |
| Progress Note:  _______________________________________________________  _______________________________________________________  _______________________________________________________  _______________________________________________________  _______________________________________________________  _______________________________________________________  _______________________________________________________  _______________________________________________________  _______________________________________________________  _______________________________________________________  _______________________________________________________  _______________________________________________________  _______________________________________________________  ____________________________________________________ | | | Laboratory results / Other comments: | | |
| Outcome:   Completely recovered   Not yet completely recovered.   Deteriorated   Permanent damage   Death   Death   Ongoing   Unknown | If resolved, date of resolution:  |___|___|/|___|___|/|___|___|  *day month year* | Investigator’s signature:  ________________________________  Date:__________________________ | | | |

# APPENDIX X1. SERIOUS ADVERSE EVENT FORM – INITIAL REPORT.

| **SERIOUS adverse even form – initial report** | | |
| --- | --- | --- |
| **1. Study**  **Number: U**|___|___|___|___|___| | **2. Day 0 Date:** |___|___|/|___|___|/|___|___|  ***day month year*** | **3. Treatment**  **Number:**  _______________ |

| Event description:______________________________________________________________________________  *(symptom, sign, or laboratory abnormality)* | | | | |
| --- | --- | --- | --- | --- |
| Date of event onset:  |___|___|/|___|___|/|___|___|___|___|  day month year | | Date event reported:  |___|___|/|___|___|/|___|___|___|___|  day month year | | Indicate reason for serious AE:   Fatal   Life-threatening   Resulted in significant /  persistent disability or  incapacity   Resulted in hospitalization   Prolonged hospitalization   Required medical / surgical  intervention to prevent serious  outcome   Other:____________________ |
| Maximum event severity:   Mild  Moderate   Severe  Life-threatening | | Maximum relationship to study drugs:   Definitely unrelated (None)   Unlikely   Possible   Probable   Definite | |  |
| Was the event unexpected?  Yes ____No____ | |  |  |  |
| Clinical history:  ________________________________________  ________________________________________  ________________________________________  ________________________________________  ________________________________________  ________________________________________  ________________________________________  ________________________________________  ________________________________________  ________________________________________  ________________________________________  ________________________________________  ________________________________________  ________________________________________  ________________________________________  ________________________________________  ________________________________________  ________________________________________  ________________________________________  ________________________________________  ________________________________________  ________________________________________ | | | Relevant past medical history:  ________________________________________  ________________________________________  ________________________________________  ________________________________________  ________________________________________ | |
|  |  |  | Concomitant medications:  1.__________________________________________  2.__________________________________________  3.__________________________________________  4.__________________________________________  5.__________________________________________ | |
|  |  |  | Action taken: (tick all that apply)   No change in current management   Study medication discontinued   Specific treatment given   Patient hospitalized   Laboratory tests obtained   Other:____________________   Other:____________________ | |
| Date form completed:  |___|___|/|___|___|/|___|___|___|___|  *day month year* | Investigator’s name (printed): ________________________________  Investigator’s signature: ______________________________________ | | | |

# APPENDIX XII. CLINICAL RECORD FORMS

| **UMSP clinical record form (1):** | | | | | | | | |
| --- | --- | --- | --- | --- | --- | --- | --- | --- |
| **Patient**  **Initials:** | **1. Study Number: U**|___|___|___|___|___| | | **2. Day 0 Date:** |___|___|/|___|___|/|___|___|  ***day month year*** | | | | **3. Treatment Number:** |___|___|___| | |
| **4. Age:_______years______months** | | **5. Gender: M _____F _____** | | | **6. Weight** *(kg):* | **7. Known drug allergies: Yes ___No ___Unknown ___**  **If yes, describe___________________________________** | | |
| ***List all medications taken within the last 2 weeks*** | | | | | | | | |
| **Drug** *(if name unknown, list by letter – “Unknown Drug A”)* **(a)** | | | | **Dose (b)** | | | | **Date last dose taken (c)** |
| 8. | | | | |  Complete  Incomplete  Unknown  N/A. | | --- | | | | |  |
| 9. | | | | | |  Complete  Incomplete  Unknown  N/A. | | --- | | | --- | --- | | | | |  |
| 10. | | | | | |  Complete  Incomplete  Unknown  N/A. | | --- | | | --- | --- | | | | |  |
| 11. | | | | | |  Complete  Incomplete  Unknown  N/A. | | --- | | | --- | --- | | | | |  |

|  | **day 0** | **day 1** | **day 2** | **day 3** | **day 7** | **day 14** | **day 21** | **day 28** | **day __** | **day __** | **day __** |
| --- | --- | --- | --- | --- | --- | --- | --- | --- | --- | --- | --- |
| **DATE** |  |  |  |  |  |  |  |  |  |  |  |
| 12. Fever in past 24h *(Y/N)* |  |  |  |  |  |  |  |  |  |  |  |
| 13. Weakness |  |  |  |  |  |  |  |  |  |  |  |
| 14. Muscle/joint aches* |  |  |  |  |  |  |  |  |  |  |  |
| 15. Headache* |  |  |  |  |  |  |  |  |  |  |  |
| 16. Anorexia |  |  |  |  |  |  |  |  |  |  |  |
| 17. Nausea* |  |  |  |  |  |  |  |  |  |  |  |
| 18. Vomiting |  |  |  |  |  |  |  |  |  |  |  |
| 19. Abdominal pain* |  |  |  |  |  |  |  |  |  |  |  |
| 20. Diarrhea |  |  |  |  |  |  |  |  |  |  |  |
| 21. Cough |  |  |  |  |  |  |  |  |  |  |  |
| 22. Pruritis |  |  |  |  |  |  |  |  |  |  |  |
| 23. Tinnutus* |  |  |  |  |  |  |  |  |  |  |  |
| 24. Behavioural changes |  |  |  |  |  |  |  |  |  |  |  |
| 25. “Flu” |  |  |  |  |  |  |  |  |  |  |  |
| 26. Other______________ |  |  |  |  |  |  |  |  |  |  |  |
| 27. Other______________ |  |  |  |  |  |  |  |  |  |  |  |
| 28. Adverse event  reported† *(Y/N)* |  |  |  |  |  |  |  |  |  |  |  |
| Initials |  |  |  |  |  |  |  |  |  |  |  |

****Only assess in children > 3 years of age. For children < 3 and those unable to answer, enter N/A.***

***† Adverse event reported if symptom is new or worsening and grade is > 2. Notify Kampala core facility immediately of all serious adverse events.***

| **UMSP clinical record form (2):** | | | |
| --- | --- | --- | --- |
| **Patient**  **Initials:** | **1. Study Number: U**|___|___|___|___|___| | **2. Day 0 Date:** |___|___|/|___|___|/|___|___|  ***day month year*** | **3. Treatment Number:** |___|___|___| |

|  | **day 0** | **day 1** | **day 2** | **day 3** | **day 7** | **day 14** | **day 21** | **day 28** | **Day __** | **day __** | **day __** |
| --- | --- | --- | --- | --- | --- | --- | --- | --- | --- | --- | --- |
| **DATE** |  |  |  |  |  |  |  |  |  |  |  |
| 29. Temperature *(ºC)* |  |  |  |  |  |  |  |  |  |  |  |
| 30. Dehydration |  |  |  |  |  |  |  |  |  |  |  |
| 31. Jaundice |  |  |  |  |  |  |  |  |  |  |  |
| 32. Chest |  |  |  |  |  |  |  |  |  |  |  |
| 33. Abdomen |  |  |  |  |  |  |  |  |  |  |  |
| 34. Skin |  |  |  |  |  |  |  |  |  |  |  |
| 35. Tablet test |  |  |  |  |  |  |  |  |  |  |  |
| 36. Other__________ |  |  |  |  |  |  |  |  |  |  |  |
| 37. Other__________ |  |  |  |  |  |  |  |  |  |  |  |
| 38. Adverse event  reported† *(Y/N)* |  |  |  |  |  |  |  |  |  |  |  |
| If abnormality noted on physical exam, describe all physical findings for the abnormal exam |  |  |  |  |  |  |  |  |  |  |  |
| **Initials** |  |  |  |  |  |  |  |  |  |  |  |

**** Follow age-based guidelines: Tablet test – > 9 mo; Heel-toe – > 2 years; Romberg – > 4 years. Answer N/A for younger children and uncooperative patients.***

***† Adverse event reported if exam sign is new or worsening and grade is > 2. Notify Kampala core facility immediately of all serious adverse events.***

| **UMSP clinical record form (3):** | | | |
| --- | --- | --- | --- |
| **Patient**  **Initials:** | **1. Study Number: U**|___|___|___|___|___| | **2. Day 0 Date:** |___|___|/|___|___|/|___|___|  ***day month year*** | **3. Treatment Number:** |___|___|___| |

|  | **day 0** | **day 1** | **day 2** | **day 3** | **day 7** | **day 14** | **day 21** | **day 28** | **day __** | **day __** | **day __** |
| --- | --- | --- | --- | --- | --- | --- | --- | --- | --- | --- | --- |
| **DATE** |  |  |  |  |  |  |  |  |  |  |  |
| 40. Parasite density  *(asexual parasites/ul)* |  |  |  |  |  |  |  |  |  |  |  |
| 41. Species |  |  |  |  |  |  |  |  |  |  |  |
| 42. Gametocyte density |  |  |  |  |  |  |  |  |  |  |  |
| 43. Haemoglobin*† *(g/dL)*  [grade] | [ ] |  |  |  |  |  |  | [ ] |  |  |  |
| Initials |  |  |  |  |  |  |  |  |  |  |  |

****(Grade on scale of 0-4: normal = 0; mild abnormality = 1; moderate = 2; severe = 3, life-threatening = 4)***

***† Any haemoglobin <*** 5g/dl measured after Day 0 is a serious AE. Notify Kampala core facility immediately of all serious adverse events.

| **RECORD OF ADDITIONAL MEDICATION GIVEN DURING STUDY** | | | | |
| --- | --- | --- | --- | --- |
| **Medication (a)** | **Indication (b)** | **Dose (c)** | **Duration (d)** | **Date started (e)** |
| **60.** |  |  |  |  |
| **61.** |  |  |  |  |
| **62.** |  |  |  |  |
| **63.** |  |  |  |  |
| **64.** |  |  |  |  |
| **65.** |  |  |  |  |
| 66. |  |  |  |  |
| **67.** |  |  |  |  |
| **68.** |  |  |  |  |

| **UMSP clinical record form (4):** | | | |
| --- | --- | --- | --- |
| **Patient**  **Initials:** | **1. Study Number:**  **U**|___|___|___|___|___| | **2. Day 0 Date:** |___|___|/|___|___|/|___|___|  ***day month year*** | **3. Treatment Number:** |___|___|___| |

| complete efficacy outcome | |
| --- | --- |
|  ETF     LCF   LPF   ACPR     N/A (Tick appropriate Incomplete Efficacy Outcome **)** | If failed, **STUDY DAY** of clinical failure (0-28) __________  **Reason for Clinical Failure:**   Severe malaria/danger signs with parasitemia Days 0-3 🡪 Specify criteria _________   Severe AE requiring change in treatment Days 0-2 🡪 Specify criteria _____________   Day 2 parasite count > Day 0 count   Parasitemia on Day 3 with temperature > 37. 5   Day 3 parasite count > 25% Day 0 count   Severe malaria with parasitemia Days 4-28 🡪 Specify criteria __________________   Parasitemia on Days 4-28 with temperature > 37. 5 OR History of fever in past 24 hours. |

**Outcome Classification**

**ETF🡪** Assessed Days 0-3

**LCF🡪** Assessed Days 4-28 and previously not an ETF.

**LPF🡪** Assessed Day 4-28

and previously not an ETF

or LCF.

**ACPR🡪** Assessed Day 28 and previously not an ETF, or LCF.

| INCOMPLETE EFFICACY OUTCOME |
| --- |
|  **Excluded** - If yes, reason for exclusion: (Must tick one reason below) Last day of follow-up _____   Other antimalarial use: if yes, describe__________________________   Withdrew informed consent   Concomitant febrile illness: if yes, diagnosis_____________________   Lost   Error/protocol violation made during follow-up that prevented outcome classification: ______________________________ |

# Appendix X1II. UMSP Adverse Record Form

| **adverse event record form** | | | |
| --- | --- | --- | --- |
| **Patient**  **Initials:** | **1. Study Number: U**|___|___|___||___||___| | **2. Day 0 Date:** |___|___|/|___|___|/|___|___|  ***day month year*** | **3. Treatment Number:** |___|___|___| |

|  | ***Complete on day first reported*** | | | ***Complete on day first reported and update as needed*** | | | ***Complete on final day*** | | |
| --- | --- | --- | --- | --- | --- | --- | --- | --- | --- |
| **Event description (a)** | **Date of event onset (b)** | **Date event reported**  **(c)** | **Initials of person reporting** | **Maximum severity* (d)** | **Maximum relationship† (e)** | **Serious? ‡ *(Y/N)***  **(f)** | **Episodic?**  ***(Y/N)***  **(g)** | **Outcome †† (h)** | **Date event resolved‡‡ (i)** |
| **80.** |  |  |  |  |  |  |  |  |  |
| **81.** |  |  |  |  |  |  |  |  |  |
| **82.** |  |  |  |  |  |  |  |  |  |
| **83.** |  |  |  |  |  |  |  |  |  |
| **84.** |  |  |  |  |  |  |  |  |  |
| **85.** |  |  |  |  |  |  |  |  |  |
| **86.** |  |  |  |  |  |  |  |  |  |
| **87.** |  |  |  |  |  |  |  |  |  |
| **88.** |  |  |  |  |  |  |  |  |  |
| **89.** |  |  |  |  |  |  |  |  |  |
| **90.** |  |  |  |  |  |  |  |  |  |
| **91.** |  |  |  |  |  |  |  |  |  |
| **92.** |  |  |  |  |  |  |  |  |  |
| **93.** |  |  |  |  |  |  |  |  |  |
| **94.** |  |  |  |  |  |  |  |  |  |

*** d) Severity:** *Rank on scale of 1-4: mild = 1; moderate = 2; severe = 3, life-threatening = 4*

**† e) Relationship:** *Rank on scale of 0-4: none = 0; unlikely = 1; possible = 2; probable = 3; definite = 4*

***‡ f) Serious:*** Criteria for serious AE: fatal, life-threatening, results in or prolongs hospitalization, results in significant or persistent disability or capacity requires medical / surgical intervention to prevent serious outcome. **If serious, report to Kampala core facility staff immediately. They will assist with patient management, assist with completion of serious AE forms, and report the AE if necessary.**

**††h) Outcome:** *Rank on scale of 1-7: completely recovered = 1; not yet completely recovered = 2; Deteriorated = 3; permanent damage = 4; Death = 5; ongoing = 6; unknown = 7.*

**‡‡ i) Date event resolved:** *Complete on Day 28 – If AE still ongoing at end of follow-up, indicate in question (h).*
