# Supplementary material for: Efficacy of Quinine, Artemether-Lumefantrine and Dihydroartemisinin-Piperaquine as Rescue Treatment for Uncomplicated Malaria in Ugandan Children
Source: PLoS One. 2013 Jan 22;8(1):e53772. doi: 10.1371/journal.pone.0053772 (PMC3551967; doi:10.1371/journal.pone.0053772)
Supplement: File S1 — InGenius Gel Documentation and Analysis system Standard Operating Procedure (SOP). (DOC) [file pone.0053772.s003.doc]

PARA_EPI_PRO_203
InGenius Gel Documentation and Analysis system

**Table of contents**

[General information](#i1)

[Responsibilities](#i2)

[Definitions and abbreviations](#i3)

[Method](#i4)

[Safety and environment](#i5)

[Attachments and forms for completion](#i6)

[Revision](#i7)

[Approval and distribution](#i8)

**1** **General information**

1.1 Aim and application

- Imaging, documenting and analysing gels are part of molecular biology research. InGenius is an advanced gel documentation and gel analysis system that fully automates molecular biology imaging. It is a fluorescent imaging system with a UV transilluminator built in a compact and dark room. It produces images of electrophoresis gels stained with Ethidium bromide.
- GeneSnap is the acquisition software specifically developed for capturing electrophoretic gel images in the InGenius system.
- GeneTools is the 1D gel and 2D spot blot analysis software that analyzes a gel from loading the image to output of results.

1.2 Legislation and standards

- According to ISO 15189 it is required that all documents relevant to the quality management system are uniquely identified; electrophoretic gel images captured with the GeneSnap software and cameras have unique identification numbers. The InGenius system is completely working under GLP conditions.

[**⮴**](#terug)

**2 Responsibilities**

- All personnel working in the genome facilities, which include the Post-PCR laboratory where the InGenius system is installed and used, are responsible for following the genome regulations and work according GLP rules.
- The heads of the different units are responsible for implementation of the rules and SOP’s.

[**⮴**](#terug)

**3** **Definitions and abbreviations**

- See DIR_COM_010 Definities en afkortingen (Definitions and abbreviations)

[**⮴**](#terug)

**4 Method**

4.1 Initializing apparatuses

- Switch on the monitor, computer processor and thermal printer
- Switch on the power of the InGenius darkroom and select the appropriate light source on the UV transilluminator (Trans UV on)
- Start the GeneSnap program by double clicking the icon on the Windows desktop
- You will see the GeneSnap User box, type into the space your name and click the OK button. Each user can enter a name or title for their own individual user preferences so that whenever you start GeneSnap and enter a particular name at the log-in stage the system adapts for that user’s preferred settings. In future you only need select your name from the drop down list.
- GeneSnap will now fully open and you will see an image window appear.

4.2 Image capturing using the GeneSnap software

- Position the stained gel on the UV transilluminator as straight as possible and close the door until the interlock clicks.

Note: You will not be able to turn your picture in the GeneTools software afterwards

- To view a “live” image of your gel, click the GREEN camera icon until it appears RED.
- Adjust the camera to maximal iris opening : adjust Aperture to 12
- Zoom in as much as possible without losing part of the gel
- Focus

- Click the saturation button on the right hand side of the GeneSnap software module

- Adjust the exposure time in the GeneSnap module, as such that you take the highest possible exposure time without saturating ANY weight marker band.

Note: Weight markers are loaded in the same amount on each gel, which is why these are used to standardize exposure time of the picture taken to limit variability. It will also to some extent compensate for different staining times and ethidium bromide solutions.

- It is important that the amount of weight marker is the same on each gel, use well calibrated pipettes for loading and preparing the weight marker; use the same type of well-former comb and standardize as much as possible the gel thickness for each analysis as this effects the final appearance of the marker.
- Do not take an exposure time of less than 40 msec. In the unlikely event that even 40 msec results in saturation, decrease the iris opening of the camera until all saturation of weight marker bands disappears.
- Alter the histogram settings randomly (left below) and click the three buttons on the right. This ensures that optimal illumination and contrast settings are used, employing all grey values from the histogram

4.3 Saving the captured image

- When selecting ‘FILE/SAVE AS’ in the file menu, the image is saved in the Syngene Secure SGD format.
- After saving, click the ‘Print’ icon to send a copy of the image to the thermal printer for a hard copy output.
- Conditions of ‘name giving’ for saving the image:
  - 1. Initials of the person who takes the picture of the gel;
    2. Underscore;
    3. Date (ddmmyyyy);
    4. The number of the gel (see molecular weight marker template), one letter for an electrophoresis in the morning, two letters for afternoon;
    5. Underscore;
    6. The type of genotyping marker with which the samples were amplified

e.g. ABC_24102008a_GLURP (first gel in the morning from GLURP electrophoresis)

e.g. ABC_24102008bb_SSP (second gel in the afternoon from SSP PCR electrophoresis)

| **Genotyping marker and family** | **Suffix** | **Name Photo** |
| --- | --- | --- |
| GLURP | GLURP | ABC_ddmmyyyya_GLURP |
| SSP | SSP | ABC_ddmmyyyya_SSP |
| MSP2: 3D7 family* | 3D7 | ABC_ddmmyyyya_3D7 |
| MSP2: FC27 family* | FC27 | ABC_ddmmyyyya_FC27 |
| MSP1: K1 family | K1 | ABC_ddmmyyyya_K1 |
| MSP1: MAD20 family | MAD20 | ABC_ddmmyyyya_MAD20 |
| MSP1: RO33 family | RO33 | ABC_ddmmyyyya_RO33 |

* For MSP2 with both families on one gel: ABC_ddmmyyya_MSP2

- When ‘FILE/EXPORT’ is selected, the image can be saved in a range of formats including TIF, BMP, GIF, PCX, TGA, WPG and JPEG. Images can then be exported to other software packages: export as Uncompressed ‘TIF’ file, check the box ‘Convert to 8 bit data’ for later use.

4.4 Image analysing using the GeneTools software

- Open the picture in the GeneTools software.
- Define the part of the picture to analyze by selecting the range of the weight marker. On top, this should be slightly above the highest marker; below it usually coincides with the edge of the gel. Left and right are set according to the lanes present.

- Set the number of lanes you expect and open the picture, which will be analyzed automatically. If you created a defined number of tracks, you can adjust the lanes manually.
- When finished, the window will show: Image (with tracks and bands marked); histogram (with peaks and edges); results (place arrow cursor over the results table and click the right mouse button to bring up the list of results fields that can be displayed and make your selections); calibration panel.
- Use the following settings in the ‘Molecular weight calibration’ menu:

- Adjust the Integration parameters as follows:

- To assign the values to a calibration marker, simply point to and click on the first band of the marker. Next select the Molecular weights icon and from the drop down menu library, chose your marker (you can edit or enter your own preferred markers).
- Make sure all the bands of the markers match the actual peaks. Delete wrongfully detected marker peaks from the marker tracks.
- The same weight marker is used on each gel for inter gel comparisons.
- Check the peak height of the 500bp band of each weight marker lane on the gel. Determine the lowest value. Adjust the Integration parameters as follows:

- Note: Setting the peak height detection threshold to 5% of the lowest 500bp marker is an arbitrary rule. It just ensures that the same peaks would be scored on all of the gels, while ignoring smaller peaks.
- Now, the following detected bands can be removed from the gel (only these bands):
- False bands in the weight marker lanes
- Bands detected that are not really bands, but come from stained impurities or dust on the gel. These artefacts are characterized by the fact that they are not spread out over the track boundaries from left to right.
- Bands well above the largest weight marker fragment. As predefined area of the gel is analyzed, these will probably not occur.
- Bands well below the smallest weight marker fragment.
- Bands at the lower and upper edge of the gel, which are caused by more intense bromide staining of the background.

**! All other bands must be retained**

- Bands can also be added manually. This is done when you see a band of which you do not believe it is an artefact, but is clearly present.
- Then assuming you have all the necessary bands shown, click the ‘Assign from standards’ button. The track is immediately calibrated and all other bands on the gel have their MW determined. These are displayed in the Results table.
- The result table is exported to an Excel file by clicking the icon on top of the window.
- Save the Excel file with the same name as the picture analyzed. Print and keep this page with the template of the electrophoresis which contains the hard copy of the picture analyzed.

4.5 Procedure double reading gels

- Double reading of gels is performed by a second qualified lab technician trained in the InGenius Gel Documentation and Analysis system.
- The second reader opens the original picture with the GeneTools software and analyzes the gel following the procedure described above in step 4.4.
- The result table is exported to an Excel file by clicking the icon on top of the window.
- The second reader saves the file as follows:

original name of the file_own initials

e.g. ABC_24102008a_GLURP_XYZ

- The second reader opens the original Excel file and copy/pastes the own reading next to the first reader’s results and saves the file under the name of the second reader’s file (see previous step).

**! Do not save the file under the first reader’s name, as this is the original data**

- Print the page with both results and add to the Electrophoresis template.
- The second reader enters the own results in the Excel worksheets next to those of the first reader and compares them.
- If there is a discrepancy in result, recrudescence is considered to be the result rather than new infection, so that analysis will continue with the next marker to exclude any uncertainty.

[**⮴**](#terug)

**5 Safety and environment**

- Always take precautions when handling gels stained with Ethidium bromide. Wear protective clothes and gloves. The gels containing Ethidium bromide are thrown away in the yellow waste containers.
- See also VWM_PRO_S011 [Werken met Ethidium bromide](http://webiso/projects/webiso/pub/VEILIGHEID_WELZIJN_MILIEU/released/VWM_PRO_S011.html)

[**⮴**](#terug)

**6 Attachments and forms for completion**

- Reference: InGenius LHR Gel Documentation and Analysis system: Installation and Image Capture Guide
- Reference: InGenius LHR Gel Documentation and Analysis system: Quick Guide-Image Analysis

[**⮴**](#terug)

7 Revision

| **Revision** | |
| --- | --- |
| **Changes with respect to the previous published version:** | Use of new template  Corrected names ascribed to photos (4.3)  Revision 25/09/2012: added procedure 2^nd^ reader |

[**⮴**](#terug)

8 Approval and distribution

| Approval and distribution | |
| --- | --- |
|  | **Name and function** |
| **Initiated by:** | Chantal Van Overmeir (Quality responsible unit Biomedical Sciences/Parasitology/Malariology) |
| **Revised by:** | Annette Erhart (Head of unit, ad interim, Biomedical Sciences/Parasitology/Malariology) |
| **Approved by:** | Jakke Van den Abbeele (Head of unit, ad interim, Biomedical Sciences/Parasitology/Malariology) |
| **Manual distribution:** | 1 copy available in the laboratory  Preferably no hard copies of this document should be made unless absolutely necessary. |

[**⮴**](#terug)
