# Supplementary material for: Efficacy of Quinine, Artemether-Lumefantrine and Dihydroartemisinin-Piperaquine as Rescue Treatment for Uncomplicated Malaria in Ugandan Children
Source: PLoS One. 2013 Jan 22;8(1):e53772. doi: 10.1371/journal.pone.0053772 (PMC3551967; doi:10.1371/journal.pone.0053772)
Supplement: File S2 — Makerere Institutional Review Board/Ethics Committee approval document. (PDF) [file pone.0053772.s004.pdf]

MAKERERE UNIVERSITY

P. O. Box 7072 Kampala, Uganda
E-mail: dean@med.mak.ac.ug

Tel: +256-414-530020
Fax: +256-414-541036/0414-532204

FACULTY OF MEDICINE
OFFICE OF THE DEAN

Friday, October 05, 2007

Dr. Ambrose Talisuna
UMSP

Dear Dr. Talisuna,

Re: **Approval of Proposal**

Your proposal entitled **"Evaluation of the best approach to retreating recurrent Malaria in Ugandan Children"** was considered and reviewed by the Research and ethics Committee. The proposal was found to be good but some amendments were suggested for incorporation in order to improve on the science of the study. I am happy the suggested changes have been incorporated.

On behalf of the committee, I am glad to inform you that the proposal has been approved. You may now proceed to carry out the research.

Please Note:

1. You are reminded that Principal Investigator is responsible for reporting any serious adverse events, requesting prior IRB (Institution Review Board) approval for all modifications, requesting continuing review and approval, and only using current IRB approved documents. You are also required to provide progress reports at regular intervals.
2. Final approval is to be granted by Uganda National Council of Science and Technology.

Yours sincerely,

Prof. Elly Katabira
Chair, Research & Ethics Committee
