# Supplementary material for: Efficacy of Quinine, Artemether-Lumefantrine and Dihydroartemisinin-Piperaquine as Rescue Treatment for Uncomplicated Malaria in Ugandan Children
Source: PLoS One. 2013 Jan 22;8(1):e53772. doi: 10.1371/journal.pone.0053772 (PMC3551967; doi:10.1371/journal.pone.0053772)
Supplement: File S3 — Uganda National Council of Science and Technology approval document. (PDF) [file pone.0053772.s005.pdf]

Uganda National Council For Science and Technology

(Established by Act of Parliament of the Republic of Uganda)

UNCST

Your Ref:...........................

Our Ref: **HS 362**

Date: **09/11/07**

Dr. Ambrose Talisuna
Assistant Commissioner Health Services
Epidemiological Surveillance
Division Ministry of HEalth
P.O Box 7272
Kampala

Dear Dr. Talisuna,

RE: RESEARCH PROJECT, "EVALUATION OF THE BEST APPROACH TO RE-TREATING RECURRENT MALARIA IN UGANDAN CHILDREN"

This is to inform you that the Uganda National Council for Science and Technology (UNCST) approved the above research proposal on **November 07, 2007.** The approval will expire on **November 07, 2008.** If it is necessary to continue with the research beyond the expiry date, a request for continuation should be made in writing to the Executive Secretary, UNCST. The UNCST has no objection to **Dr. Moses Kamya, Dr. Hasifa Bukirwa** and **Dr. Adoke Yeka** participating in the above study.

Any problems of a serious nature related to the execution of your research project should be brought to the attention of the UNCST, and any changes to the research protocol should not be implemented without UNCST's approval except when necessary to eliminate apparent immediate hazards to the research participant(s).

This letter also serves as proof of UNCST approval and as a reminder for you to submit to UNCST timely progress reports and a final report on completion of the research project.

The Resident District Commissioner of Tororo District in which the study will be conducted is informed by copy of this letter, and is kindly requested to give you the necessary assistance to accomplish the study.

Yours sincerely,

Leah Nawegulo
for: Executive Secretary
UGANDA NATIONAL COUNCIL FOR SCIENCE AND TECHNOLOGY

LOCATION / CORRESPONDENCE

Plot 3/5/7, Nasser Road
P.O. Box 6884

COMMUNICATION

TEL: (256) 414-250499, (256) 414-705500
FAX: (256) 414-234579
